# Machine learning–based inverse design for electrochemically controlled microscopic gradients of O_2_ and H_2_O_2_

**DOI:** 10.1073/pnas.2206321119

**Published:** 2022-08-01

**Authors:** Yi Chen, Jingyu Wang, Benjamin B. Hoar, Shengtao Lu, Chong Liu

**Affiliations:** ^a^Department of Chemistry and Biochemistry, University of California, Los Angeles, CA 90095;; ^b^California NanoSystems Institute, University of California, Los Angeles, CA 90095

**Keywords:** spatiotemporal heterogeneity, microwire array, O_2_ and H_2_O_2_ microenvironments, neural networks, inverse design

## Abstract

A fundamental understanding of extracellular microenvironments of O_2_ and reactive oxygen species (ROS) such as H_2_O_2_, ubiquitous in microbiology, demands high-throughput methods of mimicking, controlling, and perturbing gradients of O_2_ and H_2_O_2_ at microscopic scale with high spatiotemporal precision. However, there is a paucity of high-throughput strategies of microenvironment design, and it remains challenging to achieve O_2_ and H_2_O_2_ heterogeneities with microbiologically desirable spatiotemporal resolutions. Here, we report the inverse design, based on machine learning (ML), of electrochemically generated microscopic O_2_ and H_2_O_2_ profiles relevant for microbiology. Microwire arrays with suitably designed electrochemical catalysts enable the independent control of O_2_ and H_2_O_2_ profiles with spatial resolution of ∼10^1^ μm and temporal resolution of ∼10° s. Neural networks aided by data augmentation inversely design the experimental conditions needed for targeted O_2_ and H_2_O_2_ microenvironments while being two orders of magnitude faster than experimental explorations. Interfacing ML-based inverse design with electrochemically controlled concentration heterogeneity creates a viable fast-response platform toward better understanding the extracellular space with desirable spatiotemporal control.

## Significance

In microbiology, extracellular oxygen (O_2_) and reactive oxygen species (ROS) are spatiotemporally heterogenous, ubiquitously, at macroscopic level. Such spatiotemporal heterogeneities are critical to microorganisms, yet a well-defined method of studying such heterogenous microenvironments is lacking. This work develops a machine learning–based inverse design strategy that builds an electrochemical platform for achieving spatiotemporal control of O_2_ and ROS microenvironments relevant to microbiology. The inverse design strategy not only demonstrates the power of machine learning to design concentration profiles in electrochemistry but also accelerates the development of custom microenvironments for specific microbial systems and allows researchers to better study how microenvironments affect microorganisms in myriads of environmental, biomedical, and sustainability-related applications.

**Fig. 1.**
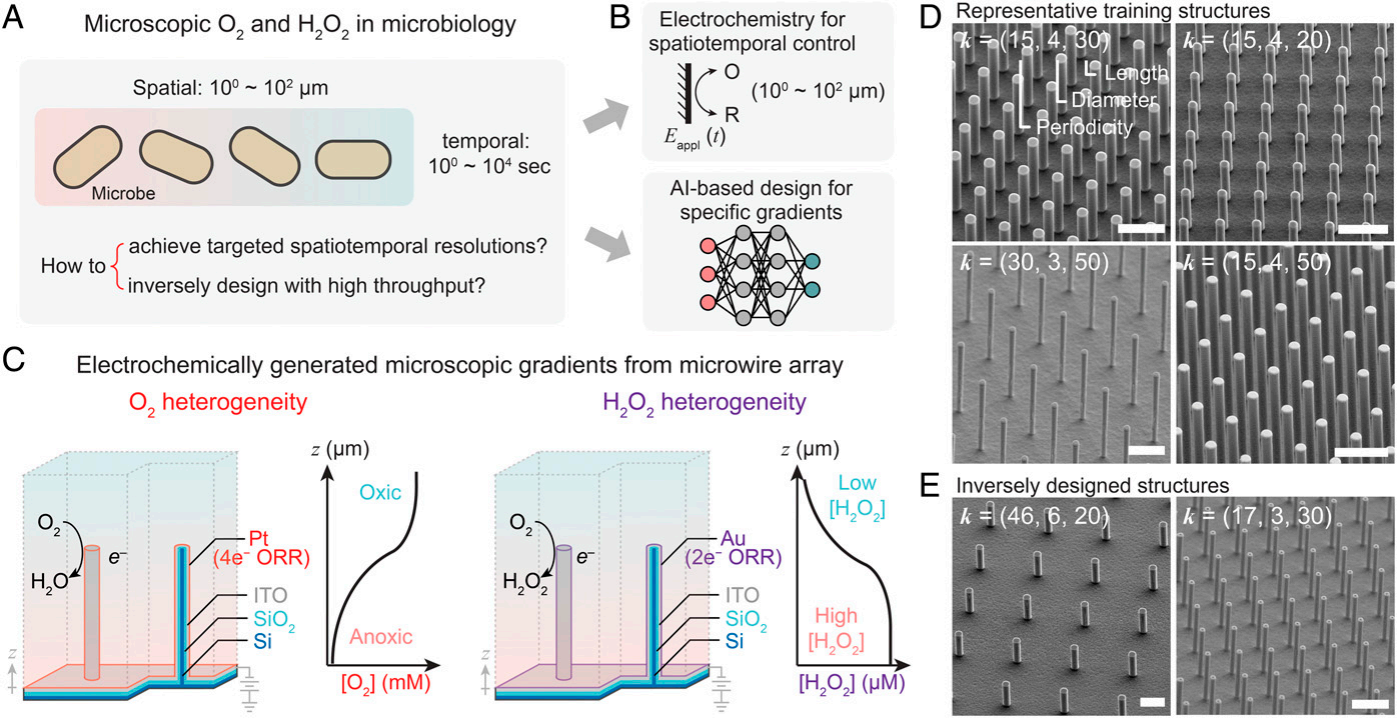
AI-based inverse design of electrochemically generated O_2_ and H_2_O_2_ heterogeneities. (*A*) The ubiquitous spatiotemporal heterogeneities of O_2_ and H_2_O_2_ in microbiology and the challenges posed in this research topic. (*B*) The combination of electrochemistry and ML-based inverse design offers a viable approach to mimicking and controlling the heterogeneities of O_2_ and H_2_O_2_ in microbiology. O, oxidant; R, reductant; *E*_appl_ (*t*), the time-dependent electrochemical voltages applied on electrodes. (*C*) The design of the electrochemically active microwire array electrodes for the generation of O_2_ and H_2_O_2_ gradients; 4e^−^ ORR & 2e^−^ ORR, four-electron and two-electron oxygen reduction reaction into H_2_O and H_2_O_2_, respectively. (*D* and *E*) The 45°-tilting images of SEM for the representative microwire arrays used for the training of the ML model (*D*) and the ones inversely designed for targeted O_2_ and H_2_O_2_ gradients (*E*); ***k*** = (*P*, *D*, *L*), the morphological vector that includes the *P*, *D*, and *L* of the synthesized wire arrays in units of micrometers. (Scale bars, 20 μm.)

Ubiquitous spatiotemporal heterogeneity of natural environments fosters the diverse and fascinating biology that our world embraces, and motivates researchers to mimic natural environments with high spatiotemporal resolution (1–5). Given their close relevance in biochemical metabolisms, dioxygen (O_2_) and hydrogen peroxide (H_2_O_2_) as a surrogate of reactive oxygen species (ROS) are two ubiquitous biologically relevant species in extracellular medium (1, 6). Their extracellular spatial and temporal distributions, particularly at the microscopic scale ranging from 1 μm to 100 μm (7–11), are critical for signal transduction, protein expression, biochemical redox balance, and regulation for cellular metabolism with extensive ecological, environmental, and biomedical implications (Fig. 1*A*) (1, 3, 8–13). A programmable creation of the spatiotemporal concentration profiles of O_2_ and H_2_O_2_ offers the freedom to mimic, control, and perturb the microenvironments of O_2_ and H_2_O_2_ and hence advance our understanding in microbiology.

Despite recent progress (14–18), there remain major technical challenges, particularly in the achievable spatiotemporal resolution and high-throughput design of concentration profiles to suit a plethora of scenarios in microbiology. Approaches based on microfluidics and hydrogels have been able to achieve concentration gradients of O_2_ and H_2_O_2_ through the provision of either O_2_/H_2_O_2_ source (14, 19–21), O_2_/H_2_O_2_ scavenging agents (15, 22, 23), or a combination of both (24) across liquid-impermeable barriers such as agar layers or polymeric thin films (25, 26). Yet such approaches, dependent on passive mass transport and diffusion across more than 10^2^ μm, are inherently incapable of achieving spatial features of less than 100 μm and temporal resolution smaller than ∼10^1^ s, the prerequisites to investigate microbiology at cluster or single-cell levels (10–12). Moreover, the large variations of extracellular O_2_ and H_2_O_2_ gradients in different microbial systems demand an inverse design strategy, which, with minimal expenditure, quickly programs a desired concentration profile catering to a specific biological scenario (2–5). The current lack of inverse design protocol impedes the adoption of controllable extracellular heterogeneity to mimic and investigate microbial systems that are of environmental, biomedical, and sustainability-related significance.

We envision that the integration of electrochemically generated concentration gradients with inverse design based on machine learning (ML) will address the aforementioned challenges (Fig. 1*B*). Electrochemistry offers a venue for transducing electric signals into microscopic concentration profiles within ∼10^0^ μm to ∼10^2^ μm away from electrodes’ surface, following the specific electrode reaction kinetics and the mass transport governing equations in the liquid phase (27). Proper designs of electrodes’ microscopic spatial arrangement and electrochemical kinetics lead to concentration gradients that are spatiotemporally programmable by time-dependent electric signals of varying voltages (28). Such benefits of electrochemically generated concentration gradients lead us to employ electrochemistry as a tool to spatiotemporally control the concentration profiles in the extracellular medium. In one example, we found that wire arrays electrochemically active toward O_2_ reduction create anoxic microenvironment about 20 μm away from the aerobic external bulk environments, modulate the size and extent of O_2_ depletion in the anoxic microenvironment by the wire array’s morphology and applied electrochemical potential (*E*_appl_), and hence enable O_2_-sensitive rhizobial N_2_ fixation in ambient air powered by renewable electricity (29). Moreover, while not reported before as far as we know, electrochemically generated concentration heterogeneity is commensurate with ML-based inverse design (30, 31), thanks to the mathematically well-defined electrochemical processes that can be numerically simulated (32, 33). We recently reported neural networks, trained by numerically simulated data, that explore the influence of electrode geometry on electrochemical N_2_ fixation and achieve optimized morphologies of wire array electrodes untenable without such an ML-based strategy (34). An inverse design for the electrochemically generated gradients will quickly program desirable microenvironments of O_2_ and ROS with high spatiotemporal resolutions, thanks to the well-reported electrochemical transformation related to O_2_ and H_2_O_2_ with high electrochemical selectivity (35, 36).

In this work, we report an inverse design based on neural networks for independent electrochemical creation of O_2_ and ROS microscopic gradients that are relevant, and mimic their extracellular heterogeneities in microbial systems. We hypothesize that careful design of electrocatalysis of O_2_ reduction reaction (ORR) can either facilitate four-electron ORR on Pt electrocatalyst for a controllable O_2_ spatiotemporal profile or promote two-electron ORR on Au electrocatalyst for a programmable generation of H_2_O_2_ gradient without significantly perturbing the O_2_ one, thanks to their concentration differences in biological mediums (∼10^−1^ μM to ∼10^1^ μM for H_2_O_2_ and ∼10^1^ μM to ∼10^2^ μM for O_2_) (2, 7–11). Electrochemically active microwire array electrodes as exemplary model systems (Fig. 1*C*) are experimentally shown to achieve tunable heterogeneities of O_2_ and H_2_O_2_ independently, with spatial resolution of ∼10^1^ μm and temporal resolution of ∼10° s, and are suitable as a platform for independently perturbing biologically relevant O_2_ and H_2_O_2_ profiles in microbial systems. We further established and experimentally validated two neural networks that inversely design the wire array electrodes’ morphologies toward targeted microenvironments of O_2_ and H_2_O_2_, respectively, which is at least one order of magnitude faster than trial-and-error numerical simulation and two orders of magnitude faster than experimental explorations. The demonstrated inverse design of electrochemically generated controlled gradients not only demonstrates a full electrochemical control of concentration profiles in an electrode’s proximity but also establishes an approach of spatiotemporally mimicking and perturbing extracellular space guided by artificial intelligence.

## Results

### Wire Array Electrodes for Electrochemical Generation of O_2_ and H_2_O_2_ Gradients.

We applied a microwire electrode array loaded with selective ORR electrocatalysts to establish customizable O_2_ or H_2_O_2_ gradients (Fig. 1*C*). Si-based microwire arrays in a square lattice were constructed through photolithography and reactive ion etching in a five-step fabrication process (see *Materials and Methods*). After thermal annealing to generate an electrically insulating oxide layer, indium-doped tin oxide (ITO) of 500 nm was deposited via sputtering near conformally onto the wire array, followed by the deposition of about 7 nm of Pt and Au for the generation of O_2_ and H_2_O_2_ gradients via selective ORR, respectively. Here the deposition of the electrically conducted ITO layer ensures a uniform distribution of the applied electrochemical potential (*E*_appl_). We employed Pt as the selective electrocatalysts of four-electron ORR (35, 37, 38) in the generation of O_2_ gradients, and employed Au for two-electron ORR (35, 39, 40) in the generation of H_2_O_2_ gradients. The morphologies (Fig. 1 *D* and *E* and *SI Appendix*, Fig. S1) and compositions (*SI Appendix*, Figs. S2 and S3) of the established wire array were characterized and confirmed by scanning electron microscopy (SEM) equipped with energy dispersive X-ray spectroscopy (EDS), with a vector ***k*** = (*P, D, L*) presenting the wire arrays’ periodicity (*P*), diameter (*D*), and length (*L*) in units of micrometers.

The prepared wire array electrodes coated with Pt and Au exhibit desirable electrochemical properties for creating O_2_ and H_2_O_2_ heterogeneities, respectively. In phosphate-buffered saline (PBS) solution, linear scan voltammograms (20 mV/s) of the deposited Pt electrocatalysts on the wire array (*SI Appendix*, Fig. S4) exhibited an onset potential of ORR at around 0.8 V vs. reversible hydrogen electrode (RHE). Linear scan voltammograms of the deposited Au electrocatalysts showed a similar onset potential of ORR at around 0.6 V vs. RHE (*SI Appendix*, Fig. S5). Experiments of rotating ring-disk electrode for the Au electrocatalysts (*SI Appendix*, Fig. S6) displayed a selectivity of H_2_O_2_ generation from O_2_ reduction up to 50% at 0.5 V vs. RHE. Thanks to the reaction–diffusion model in the electrolyte and the electrochemical boundary conditions imposed by the microwire morphology (29, 41, 42), the Pt- and Au-loaded wire array electrode transduces electric voltages *E*_appl_ into the concentration gradients of O_2_ and H_2_O_2_, respectively, at microscopic length scales.

### Electrochemical Generation and Control of O_2_ Concentration Profiles.

**Fig. 2.**
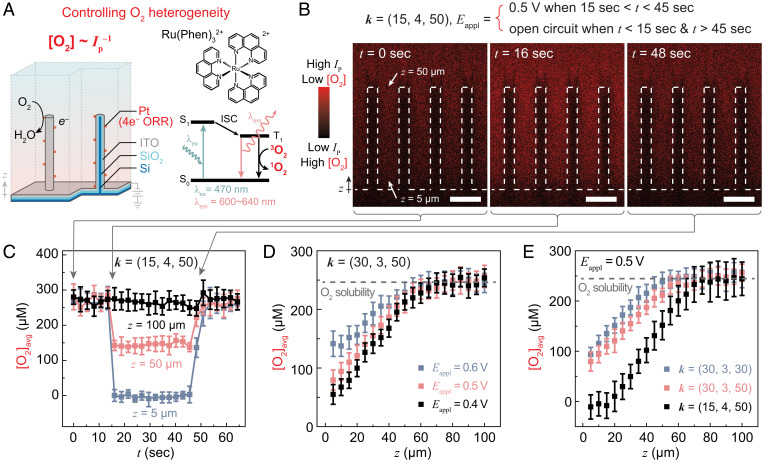
Spatiotemporal control of O_2_ gradient on Pt-loaded microwire array. (*A*) Design of Pt-loaded microwire array and the fundamentals of spatiotemporal mapping local O_2_ concentrations ([O_2_]) based on the intensity of phosphorescence emission (*I*_p_) from Tris(1,10-phenanthroline)ruthenium(II) (Ru(phen)_3_^2+^); ^3^O_2_ and ^1^O_2_, the triplet and singlet dioxygen molecules, respectively; S_0_ and S_1_, the ground state and the first excited singlet state, respectively; T_1_, the first excited triplet state; ISC, intersystem crossing; λ_ex_ and λ_em_, the wavelengths of optical excitation and emission, respectively. (*B* and *C*) Cross-sectional *I*_p_ profiles on wire array ***k*** = (15, 4, 50) at *t* = 0, 16, and 48 s (*B*) and the subsequent temporal evolution of averaged local O_2_ concentrations ([O_2_]_avg_) at different distances *z* from the base of the wire array (*C*). The values of *E*_appl_ are reported against RHE. The microwires are depicted in dashed lines in *B*. (Scale bars, 15 µm.) (*D*) Plots of [O_2_]_avg_ versus *z* under different values of *E*_appl_ for wire array ***k*** = (30, 3, 50). (*E*) Plots of [O_2_]_avg_ versus *z* in wire arrays of different ***k*** when *E*_appl_ = 0.5 V. Error bars represent SDs across multiple separate measurements in the device (*n* ≥ 3).

The Pt-deposited microwire array electrode is capable of spatiotemporally controlling the electrochemically generated O_2_ gradient. Thanks to its triplet–triplet quenching with ^3^O_2_ (43), the phosphorescence emission of Tris(1,10-phenanthroline)ruthenium(II), Ru(phen)_3_^2+^, from the intersystem crossing after optical excitation between ∼350 nm and ∼500 nm (44), was utilized to spatiotemporally probe the local concentration of O_2_ ([O_2_]) (Fig. 2*A*). The constructed microwire array electrode was housed in a homemade fluidic device (*SI Appendix*, Fig. S7) under a confocal microscope with 470-nm optical excitation (see *Materials and Methods*). Under a constant flow of aerated PBS solution with 0.15 mM Ru(phen)_3_^2+^ [[O_2_] = 0.246 mM saturated with ambient air (45)], the phosphorescence emission intensity (*I*_p_, ∼610 nm to ∼650 nm), inversely proportional to the value of [O_2_], was collected, and a calibration curve was established for the quantification of local [O_2_] values (see *Materials and Methods* and *SI Appendix*, Fig. S8). When ***k*** = (15, 4, 50) for the wire array (Fig. 2 *B* and *C*), the three-dimensional *I*_p_ mapping was recorded in a time sequence when the Pt-coated wire array was initially under an open-circuit condition (*t <* 15 s), subject to an electrochemical potential (*E*_appl_ = 0.5 V vs. RHE) from *t* = 15 and 45 s, and reverted back to the open-circuit condition when *t* > 45 s (see *Materials and Methods*). The side views of the three-dimensional *I*_p_ mapping were displayed when *E*_appl_ was initially absent (*t =* 0 s), *E*_appl_ = 0.5 V vs. RHE (*t* = 16 s), and *E*_appl_ was absent again at *t =* 48 s (“*t =* 0 sec,” *“t* = 16 sec,” and “*t* = 48 sec,” respectively, in Fig. 2*B*). The intensity of *I*_p_ was noticeably stronger within the wire array when *E*_appl_ = 0.5 V vs. RHE in comparison to the *I*_p_ values under the open-circuit conditions before and after the presence of *E*_appl_. As *I*_p_ is inversely proportional to the local values of [O_2_], this observation qualitatively suggested a decrease of [O_2_], and hence an O_2_ gradient covering the wire array region with microscopic resolution under a reductive electrochemical potential.

The averaged [O_2_] values ([O_2_]_avg_) at different distances above the bottom of the wire array *z* = 5, 50, and 100 μm were quantified and are displayed as a function of time *t* in Fig. 2*C*. While a negligible change of [O_2_]_avg_ was recorded at *z* = 100 μm (black in Fig. 2*C*), which was quite far away from the wire array, at *z* = 5 and 50 μm (blue and red, respectively, in Fig. 2*C*), significant changes of [O_2_]_avg_ up to a complete anoxic condition were observed, concurrent with the temporal presence of *E*_appl_. Such data suggest that the established O_2_ gradient can be temporally switched by electrochemical potentials faster than the temporal resolution of the confocal microscopy under the tested conditions (∼2.7 s). The electrochemically established O_2_ gradients for wire array ***k*** = (30, 3, 50) were similarly quantified at *E*_appl_ = 0.4, 0.5, and 0.6 V vs. RHE as a function of the distance above the bottom of the wire array (*z*) (black, red, and blue, respectively, in Fig. 2*D*). The steepness of the generated O_2_ gradient increased at lower *E*_appl_ values under which the electrochemical activities of O_2_ consumption on Pt were more pronounced thanks to the increased magnitude of reductive overpotential (*SI Appendix*, Fig. S4). Such an *E*_appl_-dependent O_2_ gradient showed that electrochemical input was capable of spatially controlling and yielding a desirable O_2_ gradient for potential biological applications given biologically relevant [O_2_] values and the spatial resolutions detected here (2–4, 46, 47). The electrochemically driven O_2_ gradients were also quantified at *E*_appl_ = 0.5 V vs. RHE for ***k*** = (15, 4, 50), (30, 3, 50), and (30, 3, 30) (black, red, and blue, respectively, in Fig. 2*E*). Noticeably different O_2_ gradients were observed, suggesting the capability of the wire array morphology to yield a specific O_2_ gradient. Particularly, a strictly O_2_-free local environment in aerated medium was established for ***k*** = (15, 4, 50). Such a customizable O_2_ gradient will be of interests for the study of communal interactions among microorganisms of varying degrees of O_2_ demands that are prevalent in nature (2).

### Electrochemical Generation and Control of H_2_O_2_ Concentration Profiles.

**Fig. 3.**
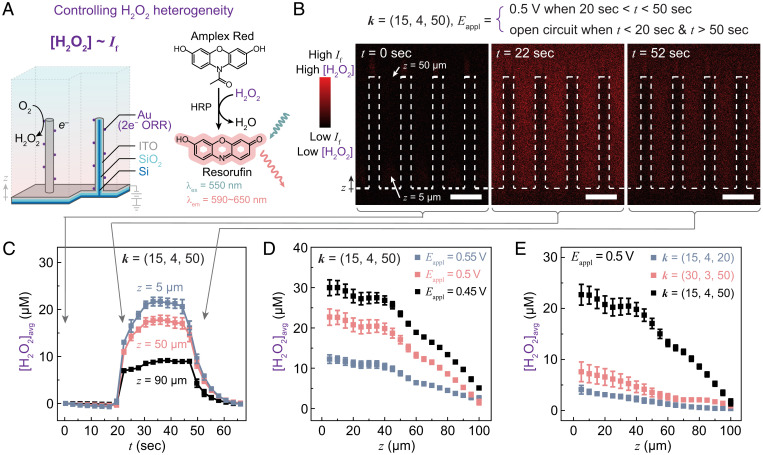
Spatiotemporal control of H_2_O_2_ gradient on Au-loaded microwire array. (*A*) Design of Au-loaded microwire array and the fundamentals of spatiotemporal mapping local H_2_O_2_ concentrations ([H_2_O_2_]) based on the intensity of fluorescence emission (*I*_f_) in the fluorogenic reaction from Amplex Red to resorufin catalyzed by HRP. (*B* and *C*) Cross-sectional *I*_f_ profiles on wire array ***k*** = (15, 4, 50) at *t* = 0, 22, and 52 s (*B*) and the subsequent temporal evolution of the averaged local H_2_O_2_ concentrations ([H_2_O_2_]_avg_) at different distances *z* from the base of the wire array (*C*). The values of *E*_appl_ are reported against RHE. The microwires are depicted in dashed lines in *B*. (Scale bars, 15 µm.) [H_2_O_2_]_avg_ = 0 when *t* = 0. (*D*) Plots of averaged local H_2_O_2_ concentrations ([H_2_O_2_]_avg_) versus *z* under different values of *E*_appl_ for wire array ***k*** = (15, 4, 50). (*E*) Plots of [H_2_O_2_]_avg_ versus *z* in wire arrays of different ***k*** when *E*_appl_ = 0.5 V. Error bars represent SDs across multiple separate measurements in the device (*n* ≥ 3).

We can similarly establish the gradients of H_2_O_2_, a potent ROS relevant to biology (48), with the use of electrochemically active wire array electrodes and H_2_O_2_-yielding Au ORR electrocatalysts. The local concentration of generated H_2_O_2_ ([H_2_O_2_]) was quantified based on the fluorogenic rection that converts nonfluorescent 10-acetyl-3,7-dihydroxyphenoxazine (Amplex Red) to fluorophore resorufin (λ_ex_ = 550 nm; λ_em_ = ∼590 to ∼650 nm) catalyzed by horseradish peroxidase (HRP) (49). Under the similar setup mentioned above (*SI Appendix*, Fig. S7), the emission intensities of resorufin (*I*_f_), and hence the local [H_2_O_2_] value, were determined for an Au-coated wire array electrode under confocal microscopy, assisted by the corresponding calibration curves (*SI Appendix*, Figs. S9–S12; see *Materials and Methods*). When ***k*** = (15, 4, 50) for the wire array (Fig. 3 *B* and *C*), the three-dimensional *I*_f_ mapping was similarly recorded in a time sequence when the Au-coated wire array was initially under an open-circuit condition (*t <* 20 s), subject to an electrochemical potential (*E*_appl_ = 0.5 V vs. RHE) from *t* = 20 and 50 s, and reverted back to the open-circuit condition when *t* > 50 s (see *Materials and Methods*). The side views of the three-dimensional *I*_f_ mapping were also displayed when *E*_appl_ was initially absent (*t =* 0 s), *E*_appl_ = 0.5 V vs. RHE (*t* = 22 s), and *E*_appl_ was absent again at *t =* 52 s (“*t =* 0 sec,” *“t* = 22 sec,” and “*t* = 52 sec,” respectively, in Fig. 3*B*). While the absence of *E*_appl_ correlates with the absence of fluorescent emission from resorufin (“*t =* 0 sec” and “*t* = 52 sec” in Fig. 3*B*), the presence of *E*_appl_ = 0.5 V vs. RHE (“*t* = 22 sec” in Fig. 3*B*) yielded significant fluorescent emission near the wire array that suggested electrochemical generation of H_2_O_2_. Meanwhile, concurring monitoring of [O_2_] suggests that the local [O_2_] are not significantly perturbed (*SI Appendix*, Fig. S13*A*), due to the relatively lower current density of ORR on the Au-based electrocatalysts under similar *E*_appl_ values (*SI Appendix*, Figs. S4 and S5). This suggests that the electrochemically controlled H_2_O_2_ gradient is nearly independent of aeration of the liquid medium, thanks to the catalytically selective generation of H_2_O_2_ and the low value of observed [H_2_O_2_] (at most, up to 30 μM) relevant for biological studies (8, 48, 50) in comparison to the air-saturated O_2_ solubility in water (246 μM) (45).

The average change of [H_2_O_2_] values ([H_2_O_2_]_avg_) at different distances above the bottom of the wire array *z* = 5, 50, and 90 μm were quantified and displayed as a function of time *t* in Fig. 3*C*. At all *z* values, the time-dependent generation of [H_2_O_2_]_avg_ measured from *I*_f_ was well correlated with the presence of *E*_appl_. A more gradual yet still relatively fast transition of the measured *I*_f_ (∼10 s), and hence [H_2_O_2_]_avg_, was observed, which was presumably due to the limited temporal resolution of the fluorogenic reaction that was needed to track local [H_2_O_2_] (51). Nonetheless, such data indicated the capability of temporally controlling the formation of H_2_O_2_ electrochemically, which can be handy as a perturbation to study the microbial response toward H_2_O_2_-based ROS (52). We also determined the electrochemically induced H_2_O_2_ gradient for wire array ***k*** = (15, 4, 50) at *E*_appl_ = 0.45, 0.5, and 0.55 V vs. RHE (black, red, and blue, respectively, in Fig. 3*D*). Significant different local accumulations of H_2_O_2_ up to 30 μM for different *E*_appl_ values were observed despite the 50-mV change of *E*_appl_. Such an observation suggests that the generated H_2_O_2_ gradient is highly sensitive and subsequently tunable by electrochemical driving forces. In addition, the morphology of wire array electrodes impacts the generated H_2_O_2_ gradient. The characterized H_2_O_2_ gradients for ***k*** = (15, 4, 50), (30, 3, 50), and (15, 4, 20) (black, red, and blue, respectively, in Fig. 3*E*) were noticeably different at the same *E*_appl_ = 0.5 V vs. RHE. The achievable range of H_2_O_2_ gradients at the microscopic level is commensurate with biologically observed ROS microenvironments (8), heralding the utility of the electrochemically generated H_2_O_2_ gradients in microbial studies.

### Establishing Neural Networks for an Inverse Design Strategy.

**Fig. 4.**
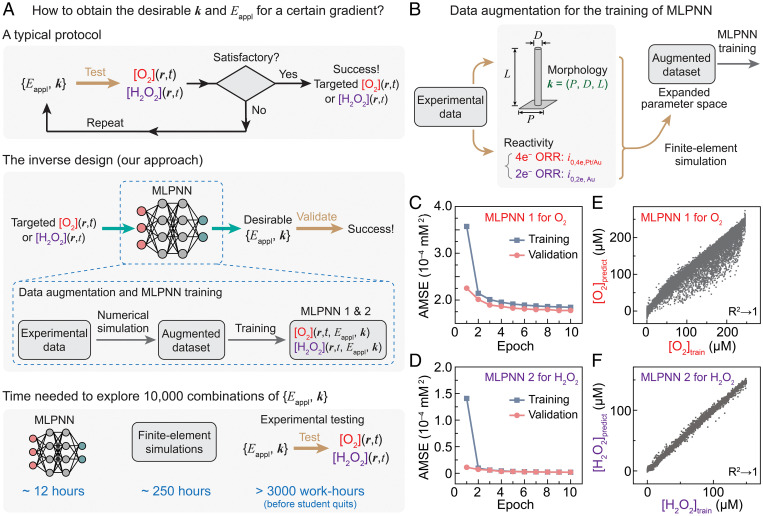
The development of inverse design for electrochemically generated O_2_ and H_2_O_2_ gradients. (*A*) Comparison between the conventional protocol and our inverse design approach for the development of suitable experimental conditions, represented as {*E*_appl_, ***k***} in order to achieve desirable spatiotemporal distributions of O_2_ and H_2_O_2_ concentrations ([O_2_](***r***, *t*) and [H_2_O_2_](***r***, *t*), respectively). MLPNN 1 & 2, multiple-layer perceptron neural networks for O_2_ and H_2_O_2_ gradients, respectively. (*B*) Protocols of data augmentation for the establishment of MLPNN; *i*_0,4e,Pt/Au_ and *i*_0,2e,Au_, the exchange current densities of four-electron and two-electron ORRs on Pt and/or Au electrocatalysts, respectively. (*C* and *D*) The AMSE in the training (blue) and validation (pink) datasets at different epochs for the gradients of O_2_ (MLPNN 1 in *C*) and H_2_O_2_ (MLPNN 2 in *D*). (*E* and *F*) Comparisons between the MLPNN-predicted values ([O_2_]_predict_ and [H_2_O_2_]_predict_) and training values ([O_2_]_train_ and [H_2_O_2_]_train_) for the local average concentrations of O_2_ (*E*) and H_2_O_2_ (*F*), respectively. R^2^, coefficient of determination.

We seek to establish computational models that can inversely predict the values of *E*_appl_ and ***k*** = (*P*, *D*, *L*) of the Pt- and Au-loaded wire array electrodes for targeted corresponding O_2_ and H_2_O_2_ gradients ([O_2_](*z*) and [H_2_O_2_](*z*)), respectively. Such an inverse design strategy for O_2_ and H_2_O_2_ microenvironments is proposed to be much more time efficient in comparison with the classical trial-and-error approach (Fig. 4*A*), and will find plentiful applications given the high variabilities of biological applications in both spatial and temporal domains (2, 3, 8). Critical inside such computational models are neural networks, trained with sufficient amounts of data, which correlate {*E*_appl_, ***k*** = (*P*, *D*, *L*)} with the [O_2_](*z*) and [H_2_O_2_](*z*) distributions. In such a regard, we employed numerical simulations based on finite element methods (FEM) (32) to augment the available data (Fig. 4*B*). FEM-based electrochemical simulations have been widely used in the understanding and design of electrochemical applications, with satisfactory accuracies (32, 33, 53–55). We established electrochemical microkinetic models that include the mass transport of redox species and the electrochemical reduction of O_2_ for the Pt and Au electrocatalysts (see *Materials and Methods*). FEM-based numerical simulations were conducted with COMSOL Multiphysics (version 5.3) for the O_2_ and H_2_O_2_ gradients near the Pt- and Au-loaded wire array electrodes, respectively. Experimental [O_2_]_avg_ and [H_2_O_2_]_avg_ values were compared with simulation results at different heights above the base of the wire array (*z*), as shown in the exemplary case when consistent results of O_2_ and H_2_O_2_ gradients were observed for ***k*** = (30, 3, 50) and *E*_appl_ = 0.5 V vs. RHE (*SI Appendix*, Fig. S13 *B* and *C*). The mean-squared errors (MSEs) between FEM-based simulations and experimental results are 9.81 × 10^−4^ mM^2^ and 4.84 × 10^−6^ mM^2^ for O_2_ and H_2_O_2_ gradients, respectively (see *Materials and Methods*). Such a consistency of results between experimental characterization and FEM-based simulations motivates us to use the augmented data from FEM-based simulations to establish neural networks to inversely predict O_2_ and H_2_O_2_ gradients.

The established neuron networks display good accuracies for the [O_2_](*z*) and [H_2_O_2_](*z*) distributions near the wire array electrodes loaded with Pt and Au electrocatalysts. We previously constructed multilayer perception neuron networks (MLPNNs) that expand the explorable parameter space of wire array electrode morphologies in electrocatalytic reduction of N_2_ (34). Here, we constructed MLPNNs that predict [O_2_](*z*) and [H_2_O_2_](*z*) based on inputs of {*E*_appl_, ***k*** = (*P*, *D*, *L*)}, which were trained based on 10,000 data points augmented from the FEM-based simulations (see *Materials and Methods*). As the model-training process proceeds with an increasing number of epochs, monotonic decreases of the average MSE (AMSE) between the training and predicted data points for the datasets of both validation and training (red and black dots, respectively) were observed in Fig. 4 *C* and *D* for the O_2_ and H_2_O_2_ gradients near Pt- and Au-loaded wire array electrodes, respectively. The fact that the values of AMSEs against the validation datasets were similar to the ones from the training datasets in Fig. 4 *C* and *D* indicates that there was no overfitting in the ML process (56). In the end, near-unity coefficients of determination (R^2^ → 1) were observed for both MLPNNs (MLPNN 1 that predicts O_2_ gradient and MLPNN 2 that predicts H_2_O_2_ gradient) (Fig. 4 *E* and *F*, respectively). The values of AMSEs from the MLPNNs reach 1.74 × 10^−4^ mM^2^ and 1.81 × 10^−6^ mM^2^ for the prediction of [O_2_](*z*) and [H_2_O_2_](*z*) based on inputs of {*E*_appl_, ***k*** = (*P*, *D*, *L*)}, respectively. Such small values of AMSEs suggest good accuracy of the developed neural networks for the inverse design of desirable O_2_ and H_2_O_2_ microenvironments.

### Exemplary Inverse Design of O_2_ and H_2_O_2_ Microenvironments Near Wire Array Electrodes.

**Fig. 5.**
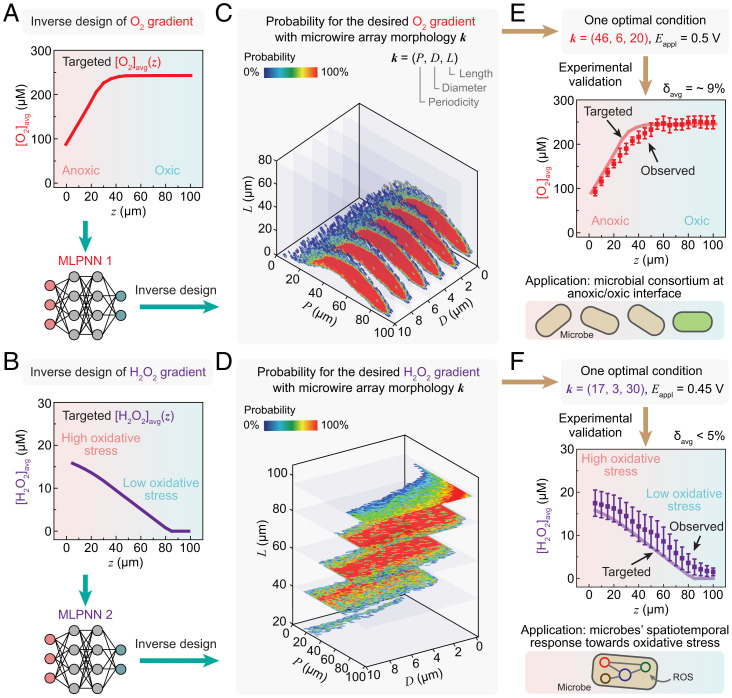
Experimental validations of MLPNN-assisted inverse design. (*A* and *B*) Targeted O_2_ and H_2_O_2_ gradients ([O_2_]_avg_(*z*) in *A* and [H_2_O_2_]_avg_(*z*) in *B*, respectively) for exemplary inverse design assisted by the developed MLPNNs. (*C* and *D*) The three-dimensional contour plots of the probabilities that MLPNN-predicted [O_2_]_avg_(*z*) (*C*) and [H_2_O_2_]_avg_(*z*) (*D*) match the targeted ones as a function of *P*, *D*, and *L* when *E*_appl_ = 0.5 V vs. RHE. (*E* and *F*) Experimental characterizations of [O_2_]_avg_(*z*) and [H_2_O_2_]_avg_(*z*) (scattered points) in comparison with the targeted ones (lines) when {*E*_appl_, ***k***} = (0.5, 46, 6, 20) in *E* and (0.45, 17, 3, 30) in *F* on Pt- and Au-loaded microwire arrays, respectively. The potential applications of those yielded gradients in microbiology are noted. Error bars represent SDs across multiple separate measurements in the device (*n* ≥ 3).

Exemplary inverse design processes with direct biological relevance were experimentally tested, with good predictabilities for the establishment of desirable O_2_ and H_2_O_2_ microenvironments. In microbiology and microbial ecology, it is desirable to establish well-defined microenvironments whose sizes are ∼20 μm to ∼100 μm in order to mimic natural heterogenous distribution of biologically relevant extracellular species such as nutrients and other microbial resources (57). Within such length scales, establishing microoxic niche (i.e., [O_2_] ≈ 100 μM) in the midst of an oxic external environment (Fig. 5*A*), prevalent in aquatic, terrestrial, and host-associated environments, is challenging yet desirable for understanding the physiology and ecology of microaerophiles and advancing our understanding of microbiomes (2); extracellular H_2_O_2_ whose concentration can achieve 15 μM (58) (Fig. 5*B*) is also of particular interest in order to study microbial sensing, communal signaling, metabolic regulation, and genetic expression toward ROS (48, 59). In such biological contexts, we aim to inversely design one O_2_ gradient (Δ[O_2_] ≈ 100 μM and Δ*z* ≈ 40 μm) and one H_2_O_2_ gradient (Δ[H_2_O_2_] ≈ 15 μM and Δ*z* ≈ 100 μm) based on our developed MLPNNs (Fig. 5 *A* and *B*). We utilized the established MLPNNs that predict [O_2_](*z*) and [H_2_O_2_](*z*) under different inputs of {*E*_appl_, ***k*** = (*P*, *D*, *L*)}, and we scored the similarity percentages between the MLPNN-predicted O_2_/H_2_O_2_ gradients and the desirable ones. Fig. 5 *C* and *D* exemplarily display the sliced mappings of similarity scores for the aforementioned O_2_ and H_2_O_2_ gradients on Pt- and Au-loaded wire arrays, respectively, as a function of ***k*** = (*P*, *D*, *L*) at *E*_appl_ = 0.5 V vs. RHE. Such multidimensional mapping, composed of 10,000 data points each in Fig. 5 *C* and *D*, showcases the parameter spaces that are predicted to yield the desirable O_2_ and H_2_O_2_ microenvironments within a certain relative uncertainty threshold (red region) (see *Materials and Methods*). It is intriguing to note that there existed multiple different wire array morphologies to yield the same desirable O_2_ and H_2_O_2_ gradients, which may not be straightforward, intuitively. We estimated that the determination of O_2_/H_2_O_2_ gradients for one parameter coordinate in the space of {*E*_appl_, ***k*** = (*P*, *D*, *L*)} will take ∼4 s for the MLPNN-based method, ∼90 s from FEM-based simulations, and ∼20 mins for the confocal characterization alone at one specific *E*_appl_ for a single wire array morphology, notwithstanding any time spent in any preceding protocols (see *Materials and Methods*). Therefore, a comprehensive exploration of the parameter space {*E*_appl_, ***k*** = (*P*, *D*, *L*)} with more than 10^4^ trials as shown above is only possible with the use of MLPNN-based inverse design, because only the MLPNN is capable of screening 10,000 parameter combinations within a reasonable amount of time in practice (∼12 h) in comparison to the ones based on FEM (∼250 h, i.e., ∼10 d) and experimental characterization (at least 3,000 work-hours without considering any practical concerns) (Fig. 4*A*).

We also conducted a spot check for the predicted O_2_ and H_2_O_2_ microenvironments by experimental validations. A Pt-based wire array electrode with ***k*** = (46, 6, 20) was picked from Fig. 5*C* as a desirable geometry, experimentally prepared (Fig. 1*E*), and experimentally tested for the established O_2_ gradient at *E*_appl_ = 0.5 V vs. RHE. Satisfactory consistency with MSE = 5.63 × 10^−4^ mM^2^ was achieved between the experimental and targeted values of [O_2_](*z*) (dots and line, respectively, in Fig. 5*E*). Similarly, an Au-based wire array electrode with ***k*** = (17, 3, 30) was picked from Fig. 5*D*, experimentally prepared (Fig. 1*E*), and experimentally tested for the H_2_O_2_ gradient at *E*_appl_ = 0.45 V vs. RHE. We also observed satisfactory consistency with MSE = 7.22 × 10^−6^ mM^2^ between the experimental and targeted values of [H_2_O_2_](*z*) (dots and line, respectively, in Fig. 5*F*). While we were unable to experimentally exhaust all of the predicted parameter space for the desirable microenvironments of O_2_ and H_2_O_2_, our experimental validations offer convincing evidence for the validity of the developed MLPNN-based inverse design for future microbiology-related research.

## Discussion

In summary, we presented a ML-based inverse design strategy for O_2_ and H_2_O_2_ concentration profiles with the use of electrochemical catalysis of ORR. We demonstrated a proof-of-concept closed-loop protocol for inversely designing O_2_ and H_2_O_2_ gradients with properly designed microwire electrodes in PBS solution, the go-to culturing medium in microbiology. By achieving concentration differences and spatial resolutions that are relevant to microbial microenvironments, the demonstrated O_2_ and H_2_O_2_ gradients are applicable for studies in microbiology. While the reported research focuses on one specific form of electrochemical boundary conditions, namely, microwire array, the reported inverse design procedures are generally applicable for any electrochemical systems that can be parameterized and analyzed by neural networks. As mass transport and concentration profiles in an electrode’s proximity are important components in electrochemistry, this work demonstrates the power of ML-based inverse design in electrochemistry. Moreover, our results will lead to a general platform that inversely designs suitable electrochemical systems for any targeted environments of O_2_ and H_2_O_2_ in microbiology. Future research will focus on the platform’s application of in vivo microbial communities and fundamental insights that can be fetched thanks to our system’s unique capabilities. Moreover, since electrochemistry is capable of modulating any redox active species such as extracellular Fe(II)/(III) species (60), as well as other extracellular metabolites sensitive to oxidative stress, such as pyocyanin (61–63), our inverse design approach based on electrochemistry is capable of controlling the other microenvironments beyond O_2_ and ROS and is generally applicable in the study of ubiquitous microenvironments in extracellular medium.

## Materials and Methods

### Chemical and Materials.

ITO-coated glass slides (06499-AB) were purchased from SPI Supplies(∼30 Ω/◻ to ∼60 Ω/◻, 22 × 40 mm). Silver (Ag) paste (16040-30) was purchased from Ted Pella. Si wafers (*p*-type, boron-doped, <100>, ∼1 Ω⋅cm^−1^ to ∼10 Ω⋅cm^−1^) were purchased from University Wafer, Inc. Platinum (Pt) wires (CHI 115) and glassy carbon electrodes (CHI 104, diameter = 3 mm) were purchased from CH Instruments. Unless specially mentioned, all chemicals and materials were purchased from Sigma-Aldrich or VWR.

### Experimental Establishment of Testing Platform.

Modified from a previously published protocol (29), the experimental testing platform as shown in *SI Appendix*, Fig. S7 consists of a fluidic cell with a three-electrode system, in order to electrochemically generate desirable gradients of O_2_ and H_2_O_2_. As the working electrode in the setup, microwire array electrodes were fabricated similarly as previously described (29). The microwire arrays were fabricated by photolithography with the use of the deep reactive-ion etching process (DRIE). After treatment in hexamethyldisilazane vapor for 10 min, precleaned Si wafers were coated with photoresist (MicroChemicals, AZ5214E; 3000 rpm spin coating) by soft baking (100 °C for 75 s), exposed in the hard contact mode by a contact aligner (Carl Suss MA6), hard baked (120 °C, 5 min), and developed to generate the periodic patterns for the array (mixture of MicroChemicals AZ400K water, 1:4 volume ratio). After creation by DRIE (Unaxis Versaline FDSE III), microwire arrays of desirable lengths were annealed under ambient air at 1,050 °C for 9.5 h to yield the insulating oxide layer, coated by 500-nm ITO through reactive sputtering (Denton Discovery 550 sputtering System), and finally deposited with a 7-nm layer of Pt or Au by an Anatech Hummer 6.2 sputtering system. The structure of the wire array was examined by SEM (ZEISS Supra 40VP SEM), and the element distribution was examined by SEM (JEOL JSM-6700F) equipped with EDS (Ametek). Patterned ITO-coated glass slides were used as the counter electrodes in the established testing platform, after the selective removal of ITO by 6 M HCl in undesirable areas on the glass slides. Ag paint as the pseudoreference electrode was applied on select areas of the ITO-coated slides so as to cover a 5 mm × 5 mm square with a layer of silver, serving as the reference electrode, while Pt was deposited on other ITO-coated areas for the creation of a counter electrode in the setup. An optically transparent fluidic cell of 250-µm height was constructed by assembling the microwire array electrodes with the prefabricated ITO glass slides, while a Gamry Interface 1010B potentiostat was used to enforce the electrochemical driving force. The setup was mounted on an inverted laser confocal microscope (Leica SP8 SMD) with sufficient working distance (680 µm), and a syringe pump was used to maintain a fixed liquid flow rate. As we particularly ensure the accuracy of applied electrochemical potentials, cyclic voltammetry in a standard ZoBell’s solution (3.3 mM K_3_Fe(CN)_6_, 3.3 mM K_4_Fe(CN)_6_, and 0.1 M KCl, 0.43 V vs. standard hydrogen electrode) was employed to calibrate the electrochemical potential of an Ag-based pseudoreference electrode as shown in *SI Appendix*, Fig. S14 (64, 65). The Ag-based pseudoreference electrode was calibrated as 0.75 V vs. RHE in PBS solution at pH = 7.4.

### Electrochemical Characterization of the Deposited Electrocatalysts.

While the deposited Pt electrocatalysts have been characterized in our prior report (29), experiments were conducted to analyze the electrocatalytic activities of ORR for the coated Au electrocatalysts. An experiment of a rotating ring-disk electrode (Pine Research, Inc., AFE6R1PT) was conducted in PBS solution using a setup with a Pt-wire counter electrode, Ag/AgCl (1M KCl) reference electrode and a modulated speed rotator (Pine Research, Inc., AFMSRCE). While a Pt ring electrode was kept at 1.9 V vs. RHE, linear scan voltammograms (20 mV/s) were recorded between 0.1 and 1.1 V vs. RHE with different rotating speeds (100, 225, 400, 625 , 900, 1,225, 1,600 pm, and 2,025 rpm) in electrolytes saturated with O_2_ and N_2_, respectively. The measurements in N_2_-saturated solution were used as the signal background.

### Quantification and Calibration of O_2_ Concentration Profiles.

Aerated PBS solution consisting of 150 µM Ru(phen)_3_Cl_2_ solution was prepared in the dark and fed into the assembled testing platform at a flow rate of 0.8 mL/min. The phosphorescence intensity mapping under confocal microscopy was measured in a 1-min time sequence. During the 1-min confocal microscopy measurement, programmed 30-s electrolysis was performed with a particular potential on the working electrode from *t* = 15 s to *t* = 45 s. The excitation wavelength was set as 470 nm, and we gathered emission intensity from 600 nm to 640 nm as phosphorescence emission intensity *I*_p_. We defined the phosphorescence emission intensity with no potential applied as *I*_0_. Normalized phosphorescence emission intensity *I*_pn_ was defined as $I_{pn}=I_{p}/I_{0}$. The phosphorescence emission intensity distribution was further translated into the concentration profiles, based on the linear relationship between O_2_ concentration and the inverse of *I*_pn_ (noted as *I*_pn_^−1^) that was experimentally determined. Ru(phen)_3_Cl_2_–containing PBS solutions of different O_2_ concentrations, ranging from [O_2_] = 25 µM to 375 µM, were prepared by bubbling a N_2_/O_2_ mixture of tunable ratio through the solution, and were pumped into the assembled fluidic device (0.8 mL/min) for calibration.

### Quantification and Calibration of H_2_O_2_ Concentration Profiles.

Aerated PBS solution consisting of 0.2 U/mL HRP and 120 µM Amplex Red (1× working solution) was prepared in the dark and fed into the assembled testing platform at a flow rate of 0.8 mL/min. The fluorescent intensity mapping under confocal microscopy was conducted in a 1-min time sequence. During the 1-min confocal microscopy measurement, programmed 30-s electrolysis with a particular potential on the working electrode from *t* = 20 s to *t* = 50 s. The excitation wavelength was set as 550 nm, and we gathered emission intensity from 590 nm to 650 nm as fluorescence emission intensity *I*_f_. The fluorescence emission intensity distribution was further translated into the concentration profiles, based on the corresponding calibration curves. In the experiments of calibrating H_2_O_2_ concentrations, darkly prepared PBS solution consisting of 0.4 U/mL HRP and 240 µM Amplex Red was combined with PBS solutions of different H_2_O_2_ concentrations, ranging from 5 µM to 60 µM, and was pumped into the assembled devices for the measurement of confocal microscopy. We found that the H_2_O_2_-induced fluorescence intensity *I*_f_ is also dependent on the specific morphologies of the wire array (***k***) and the distance away from the bottom of the wire array (*z*) (*SI Appendix*, Fig. S15), owing to the scattering and, possibly, optical absorption of the wire array electrodes (66–68). Therefore, individual calibration curves were determined for every *z* location in wire arrays with all possible ***k*** values (*SI Appendix*, Figs. S9–S12). Specific *I*_f_ correction was made to compensate the difference between the calibration experiment and gradient optical detection due to practical restrictions (*SI Appendix*, *Supplementary Text* and Fig. S16).

### FEM-Based Numerical Simulations for O_2_ Gradient and H_2_O_2_ Gradients.

FEM simulation of both O_2_ gradient and H_2_O_2_ gradient was achieved in “electroanalysis” module in COMSOL Multiphysics (Version 5.3).

#### Geometry description.

The shape of wire was represented by a column with diameter *D* and length *L*. We located each wire in the center of a cuboid of *P* × *P* × 200 µm, and the difference between the cuboid and column was geometrically defined for the electrolyte. For each point in the electrolyte, if its distance from the top of the wire not smaller than the diffusion distance, $d_{D}$, we considered that it belonged to the bulk electrolyte in which [O_2_] = 246 µM, independent of time. The boundary surface was defined as the area of which the distance to the top of the wire is $d_{D}$. On the boundary surface, the [O_2_] was the same as that in the bulk. A periodic boundary condition was applied to describe the wire array. The value of $d_{D}$ was set as 20 µm for O_2_ gradient simulation on Pt-loaded wire array electrodes (29). For O_2_ gradient and H_2_O_2_ gradient simulation on Au-loaded wire array electrodes, $d_{D}$ was measured as 50 µm (*SI Appendix*, Fig. S17).

#### Transport properties.

The diffusion of oxygen and hydrogen peroxide was simulated based on the following Eqs. **1** and**2**. $D_{O_{2}}$ and $D_{H_{2}O_{2}}$ are the diffusion coefficients of oxygen and hydrogen peroxide in aqueous solution, which were 2.2 × 10^−9^ m^2^/s and 1.5 × 10^−9^ m^2^/s respectively.

[1] $$\frac{\partial [O_{2}]}{\partial t}=D_{O_{2}}\nabla^{2}\left[O_{2}\right]$$

[2] $$\frac{\partial [H_{2}O_{2}]}{\partial t}=D_{H_{2}O_{2}}\nabla^{2}\left[H_{2}O_{2}\right].$$

#### Electroanalysis.

The potential window of *E*_appl_ is from 0.6 V vs. RHE to 0.2 V vs. RHE.

On the surface of Pt-loaded wire array electrodes, 4e-ORR took place within the potential window.

$${\text{O}}_{2}+{\text{4H}}^{+}+{\text{4e}}^{-}\to {\text{2H}}_{2}\text{O}.$$

On the electrode surface of Au-loaded wire array electrodes, two-electron and four-electron ORR reactions (2e-ORR and 4e-ORR, respectively) took place at the same time as potential-dependent selectivity.

$$\begin{array}{l} \text{2e}-\text{ORR}:{\;\text{O}}_{2}+{\text{2H}}^{+}+{\text{2e}}^{-}\to {\text{H}}_{2}{\text{O}}_{2}\\ \text{4e}-\text{ORR}:{\;\text{O}}_{2}+{\text{4H}}^{+}+{\text{4e}}^{-}\to {\text{2H}}_{2}\text{O} \end{array}.$$

On the surface of Pt-loaded wire array electrodes, the supply–consumption equilibrium was simulated as Eqs. **3** and **4** in the formalism of concentration-dependent Tafel kinetics (27),

[3] $$i_{\mathrm{loc}}=i_{4e}=-i_{0,4e,Pt}\frac{\left[O_{2}\right]}{C_{O_{2}}}exp\left(\frac{-\alpha_{c}F\eta_{Pt}n_{4e}}{RT}\right)$$

[4] $$J_{O_{2}}=\;\frac{i_{\mathrm{loc}}}{4F}.$$

Here $i_{4e}$ denotes the current density of 4e-ORR, $i_{0,4e,Pt}$ denotes the exchange current density of 4e-ORR, $i_{\mathrm{loc}}$ denotes the local current density of four-electron reduction of O_2_ on the electrode surface, $J_{O_{2}}$ denotes the local flux of O_2_ consumption from electrolyte, and $\eta_{Pt}$ is the overpotential that is defined as the difference between *E*_appl_ and the standard redox potential of O_2_/H_2_O, $E_{O_{2}/H_{2}O}^{0}$ (1.23 V vs. RHE). The $\alpha_{c}$ equals 0.5 as the transfer coefficient, *F* denotes the Faraday constant, *R* is the gas constant, *T* is the temperature, and $n_{4e}$ is the electron transfer number before the rate-determining step of 4e-ORR, of which the value is one (*SI Appendix*, Fig. S4). The local oxygen concentration is denoted as [O_2_]. $C_{O_{2}}$ is the oxygen concentration in air-saturated water at *T*. The above equation follows the textbook equations that account for the mass transport and chemical stoichiometry at electrode interfaces (27). On Pt-loaded wire array electrodes, $i_{0,4e,Pt}$ is found to be 3.0 × 10^−6^ A/m^2^ (*SI Appendix*, Fig. S4).

On the electrode surface of Au-loaded wire array electrodes, the supply–consumption equilibrium was simulated as Eqs. **5–7**.

[5] $$i_{\mathrm{loc}}=i_{4e}+i_{2e}=-i_{0,4e,Au}\frac{\left[O_{2}\right]}{C_{O_{2}}}exp\left(\frac{-\alpha_{c}F\eta_{Au}n_{4e}}{RT}\right)-i_{0,2e,Au}\frac{\left[O_{2}\right]}{C_{O_{2}}}exp\left(\frac{-\alpha_{c}F\eta_{Au}n_{2e}}{RT}\right)$$

[6] $$J_{O_{2}}=\frac{i_{4e}}{4F}+\frac{i_{2e}}{2F}$$

[7] $$J_{H_{2}O_{2}}=-\frac{i_{2e}}{2F}.$$

While most of the definitions of variables in the case of Au-loaded wire array electrodes with Eqs. **5****–7** are the same as the Pt-loaded case in Eqs. **3** and **4**, we noted that $i_{\mathrm{loc}}$ instead stands for the local current density of both two-electron and four-electron reduction of O_2_ on the electrode surface, $i_{2e}$, the current density of 2e-ORR, and $n_{2e}$ is electron transfer number before the rate-determining step of 2e-ORR, of which the value is 0.7 (*SI Appendix*, Fig. S5). On Au-loaded wire array electrodes, the catalysis selectivity of Au toward 4e-ORR and 2e-ORR is dictated by the exchange current densities $i_{0,4e,Au}$ and $i_{0,2e,Au}$, respectively. The $i_{0,4e,Au}$ is 2.0 × 10^−8^ A/m^2^, and $i_{0,2e,Au}$ is 8.0 × 10^−7^ A/m^2^, based on literature and measurement (*SI Appendix*, Fig. S5) (69, 70).

Based on a comparison with experimental gradients, the AMSE of O_2_ gradient simulation and H_2_O_2_ gradient simulation on Pt-loaded wire array electrodes and Au-loaded wire array electrodes is 9.81 × 10^−4^ mM^2^ and 4.84 × 10^−6^ mM^2^, respectively. The range of *E*_appl_ was set within the ORR potential windows, from 0.6 V vs. RHE to 0.2 V vs. RHE. Besides, a three-dimensional block was defined in the space of (*P*, *D*, *L*) as the range of morphology, from *P* = 1 µm to 100 µm, *L* = 1 µm to 150 µm, and *D* = 0.2 µm to 10 µm. By using Simulink in Matlab, we were able to generate random ***k*** value within the morphology space and calculate the corresponding gradient curves. To fulfill the ML functions, gradient profiles under 10,000 experimental conditions were included in the dataset for each developed multiple-layer perceptron neural network, respectively.

### Model Selection and Training.

The implementation of all ML code was done on a MacBook Pro with a 1.4 GHz quad-core Intel Core i5 processor and 8GB of RAM, with code specifically deployed using the JupyterLab Notebook, a Python-based programming platform widely used in data science and ML (34). In this paper, we selected MLPNNs as the ML model for inverse design. We use FEM-simulated gradients to develop the MLPNNs. Gradient data were imported from FEM results in the form of .csv files and combined into a library of data. Prior to the model development, a random 20% of the data were split from the whole dataset for later model validation. The rest of the data were split into training data and validation data, of which the percentages were 65% and 15%, respectively. Multiple cycles of model training, each cycle termed as one epoch, were conducted in order to develop the targeted MLPNNs. In each epoch, MLPNNs will be trained from the training data, followed by a validation process in the validation dataset used to provide estimates of final model accuracy after each round of training. The total ML process will include 10 epochs of forward and backward propagations. The accuracies of the developed MLPNNs model as a function of epoch numbers are plotted in Fig. 4 *C* and *D*.

As described in the section above, the training dataset was a collection of concentration gradients under different *E*_appl_ and ***k*** values. We used AMSE and SD, defined in more detail below, from Eqs. **8****–11** to quantify the ability of ML models to correctly reveal the connection between gradient curves and the two impacting factors, *E*_appl_ and ***k***. In model selection, we selected multiple-layer perceptron neural networks for gradient prediction due to the low AMSE and SD value gradient prediction (*SI Appendix*, Fig. S18).

For both O_2_ and H_2_O_2_ gradients, the predicted curve included 20 local concentration datum along the wire array from *Z* = 5 µm to 100 µm every 5 µm. *Z* is defined as the distance from the bottom of the wire array. In O_2_ gradient prediction, MSE and SD are defined as the following equations:

[8] $$MSE_{O}=\frac{1}{20}\overset{20}{\underset{n=1}{\sum }}{\left({\left[O_{2}\right]}_{\mathrm{NNs}}-{\left[O_{2}\right]}_{\mathrm{real}}\right)}^{2}$$

[9] $$SD_{O}=\sqrt{\frac{1}{N}\overset{N}{\underset{n=1}{\sum }}{\left(MSE_{O}{}_{n}-\mathrm{AMS}E_{o}\right)}^{2}}.$$

In Eqs. **8** and **9**, ${\left[O_{2}\right]}_{\mathrm{NNs}}$ stands for the predicted oxygen concentration from MLPNNs predicting the O_2_ gradient on the Pt-loaded wire array. ${\left[O_{2}\right]}_{\mathrm{real}}$ is the oxygen concentration in the simulation dataset. *MSE_O_* is defined based on the average square of concentration difference over the whole gradient profile. $\mathrm{AMS}E_{o}$ is the average MSE over the whole dataset. We calculated the SD over data under a wide range of experimental conditions to evaluate the overall precision of predictions from MLPNNs predicting the O_2_ gradient on the Pt-loaded wire array.

MSE and SD in the H_2_O_2_ gradient were defined in a similar pattern. In Eqs. **10** and **11**, ${\left[H_{2}O_{2}\right]}_{\mathrm{NNs}}$ stands for the predicted hydrogen peroxide concentration from MLPNNs predicting the H_2_O_2_ gradient on the Au-loaded wire array. ${\left[H_{2}O_{2}\right]}_{\mathrm{real}}$ is the hydrogen peroxide concentration in the simulation dataset. $MSE_{H}$ is defined based on the average square of concentration difference over the whole gradient profile. $\mathrm{AMS}E_{H}$ is the average MSE over the whole dataset.

[10] $$MSE_{H}=\frac{1}{20}\overset{20}{\underset{n=1}{\sum }}{\left({\left[H_{2}O_{2}\right]}_{\mathrm{NNs}}-{\left[H_{2}O_{2}\right]}_{\mathrm{real}}\right)}^{2}$$

[11] $$SD_{H}=\sqrt{\frac{1}{N}\overset{N}{\underset{n=1}{\sum }}{\left(MSE_{H}{}_{n}-\mathrm{AMS}E_{H}\right)}^{2}}.$$

### Morphology Prediction for Desired O_2_ Gradient and H_2_O_2_ Gradient.

In morphology prediction for the desired O_2_ gradient, we assigned *E*_appl_ = 0.5 V vs. RHE. Initially, MLPNNs predicting the O_2_ gradient on the Pt-loaded wire array randomly selected one morphology in the morphology space and calculated the corresponding O_2_ gradient. The similarity score between the calculated O_2_ gradient and target O_2_ gradient, $S_{O}$, was quantified by Eq. **12**. In the prediction process, the neural networks would find out the top 10,000 morphologies with the highest $S_{O}$ values. In Eq. **12**, ${\left[O_{2}\right]}_{T}$ is the local oxygen concentration in the target O_2_ gradient, and ${\left[O_{2}\right]}_{\mathrm{NNs}}$ is the calculated local oxygen concentration. The similarity score, $S_{O}$, is the average relative error among a collection of different *z* values, $C_{O}$. $C_{O}=\left[5,\;10,\;15,\;25,\;35,\;45,\;70\right]$, where the unit is micrometers.

[12] $$S_{O}=1-100\text{\%}\times \frac{1}{7}\overset{}{\underset{z\in C_{O}}{\sum }}\frac{\left|{\left[O_{2}\right]}_{\mathrm{NNs}}-{\left[O_{2}\right]}_{T}\right|}{{\left[O_{2}\right]}_{T}}.$$

In morphology prediction for the desired H_2_O_2_ gradient, we assigned *E*_appl_ = 0.45 V vs. RHE. Initially, MLPNNs predicting the H_2_O_2_ gradient on the Au-loaded wire array randomly selected one morphology in the morphology space and calculated the corresponding H_2_O_2_ gradient. The similarity score between the calculated H_2_O_2_ gradient and target H_2_O_2_ gradient, $S_{H}$, was quantified by Eq. **13**. In the prediction process, the neural networks would find out the top 10,000 morphologies with the highest $S_{H}$ values. In Eq. **13**, ${\left[H_{2}O_{2}\right]}_{T}$ is the local hydrogen peroxide concentration in the target H_2_O_2_ gradient, and ${\left[H_{2}O_{2}\right]}_{\mathrm{NNs}}$ is the calculated local hydrogen peroxide concentration. The similarity score, $S_{H}$, is the average relative error among a collection of different *z* values, $C_{H}$. $C_{H}=[5,\;20,\;30,\;40,\;55,\;70,\;80]$, where the unit is micrometers.

[13] $$S_{H}=1-100\text{\%}\times \frac{1}{7}\overset{}{\underset{z\in C_{H}}{\sum }}\frac{\left|{\left[H_{2}O_{2}\right]}_{\mathrm{NNs}}-{\left[H_{2}O_{2}\right]}_{T}\right|}{{\left[H_{2}O_{2}\right]}_{T}}.$$

In the sliced mapping of the H_2_O_2_ similarity score, the similarity score at *L* = 5 µm was the average of from *L* = 0 µm to 10 µm. The similarity score at *L* = 20 µm was the average of from *L* = 10 µm to 30 µm. The similarity score at *L* = 40 µm was the average of from *L* = 30 µm to 50 µm. The similarity score at *L* = 60 µm was the average of from *L* = 50 µm to 70 µm. The similarity score at *L* = 80 µm was the average of from *L* = 70 µm to 90 µm. The similarity score at *L* = 95 µm was the average of from *L* = 90 µm to 100 µm.

## Supplementary Material

Supplementary File

## Data Availability

All study data, except the code for the neural networks, are included in the article and/or *SI Appendix*. The code for the neural networks is available on B.B.H.’s GitHub account http://github.com/bbhoar/O2_H2O2_ML_PNAS.
