# Supplementary material for: Machine learning–based inverse design for electrochemically controlled microscopic gradients of O2 and H2O2
Source: Proc Natl Acad Sci U S A. 2022 Aug 1;119(32):e2206321119. doi: 10.1073/pnas.2206321119 (PMC9371721; doi:10.1073/pnas.2206321119)
Supplement: Supplementary File [file pnas.2206321119.sapp.pdf]

Supplementary Information for

Machine-learning-based inverse design for electrochemically controlled microscopic gradients of O_2 and H_2O_2

Yi Chen, Jingyu Wang, Benjamin B. Hoar, Shengtao Lu, Chong Liu*

* Corresponding author: Chong Liu

Email: chongliu@chem.ucla.edu

This PDF file includes:

Supplementary text
Figures S1 to S18
Table S1

Supplementary Text

Fluorescence intensity correction in H_2O_2 concentration calibration experiment In the H_2O_2 concentration calibration experiment, H_2O_2 solution mixed with 2× working solution (PBS solution consisting of 0.4 U/mL HRP and 240 μM Amplex Red) in a mixing tee (IDEX, U-466). In the H_2O_2 gradient measurement experiment, the mixing area was the working electrode area. In the calibration experiment setup, it required extra time for liquid to travel from mixing tee to the working electrode resulting in higher fluorescence emission intensity in calibration experiments under the same H_2O_2 concentration. The relationship between increased fluorescence intensity and extra time was revealed by measuring the I_f difference between the upstream and downstream of the wire array electrode (Fig. S16). In each calibration experiment, I_f was corrected based on the relationship.

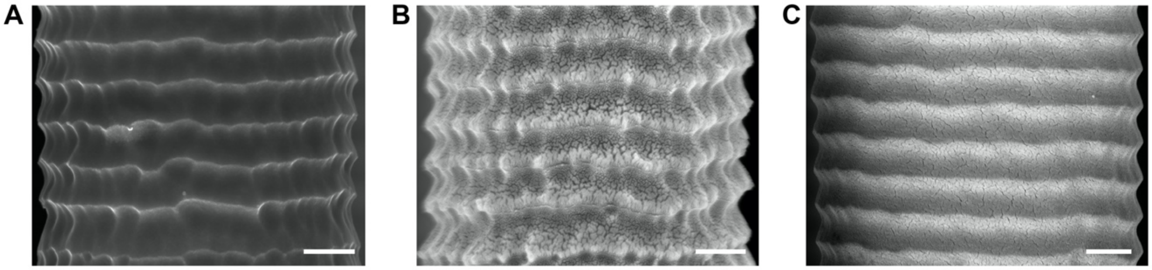

Fig. S1. Scanning electron microscopy (SEM) images for the surface of wire array electrode without the deposition of Au/Pt catalysts (*A*), loaded with Pt electrocatalyst (*B*), and loaded with Au electrocatalyst (*C*). Scale bar, 500 nm.

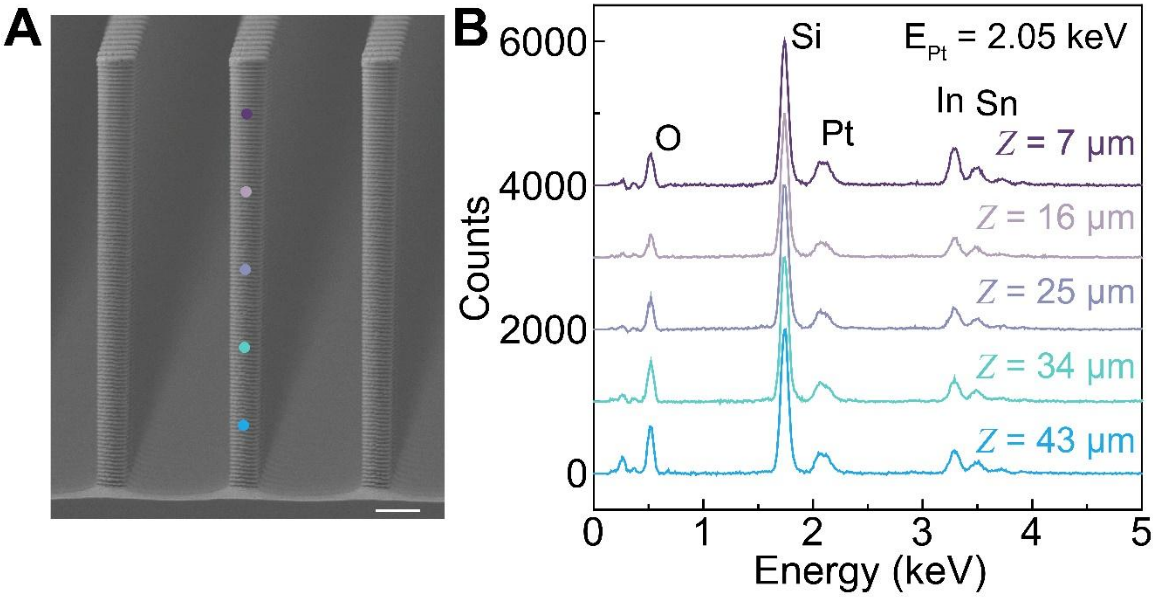

Fig. S2. Morphological characterization of Pt-coated wire array electrodes with $\boldsymbol{k}$ = (15, 4, 50) (*A*) The cross-sectional scanning electron microscopy (SEM) image depicting the side view of wire array with $\boldsymbol{k}$ = (15, 4, 50) (*B*) Energy-dispersive X-ray spectroscopy (EDS) spectra taken at different height along the Pt-coated wire array in (*A*). A homogenous distribution of Pt, In and Sn was observed along the prepared microwire array. Scale bar, 5 µm.

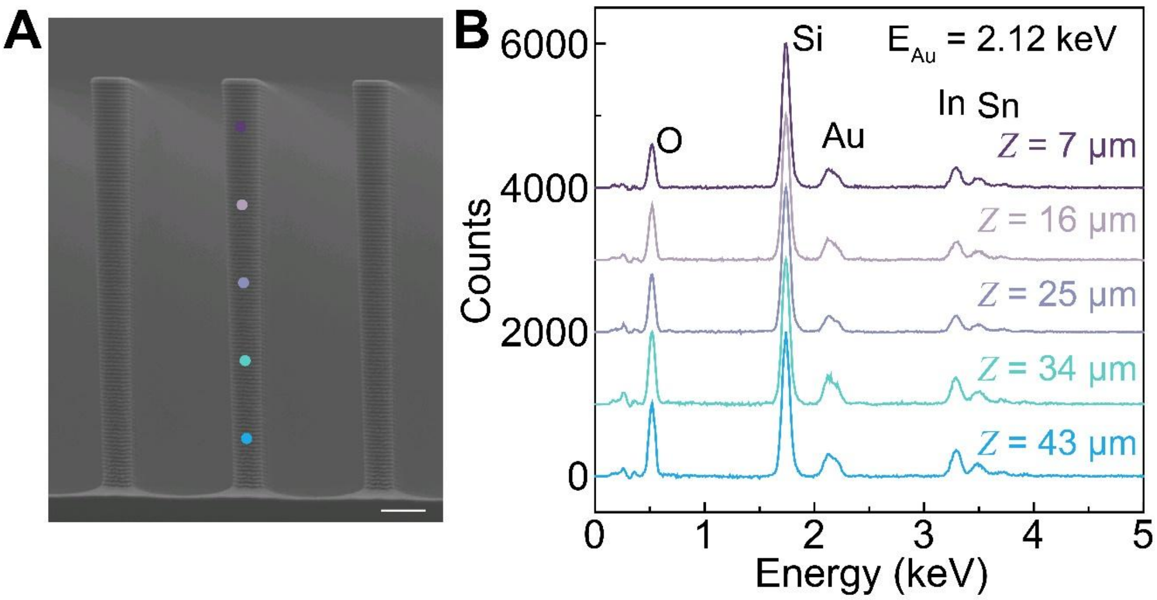

Fig. S3. Morphological characterization of Au-coated wire array electrodes with ***k*** = (15, 4, 50) (*A*) The cross-sectional scanning electron microscopy (SEM) image depicting the side view of wire array with ***k*** = (15, 4, 50) (*B*) Energy-dispersive X-ray spectroscopy (EDS) spectra taken at different height along the Pt-coated wire array in (*A*). A homogenous distribution of Au, In and Sn was observed along the prepared microwire array. Scale bar, 5 μm.

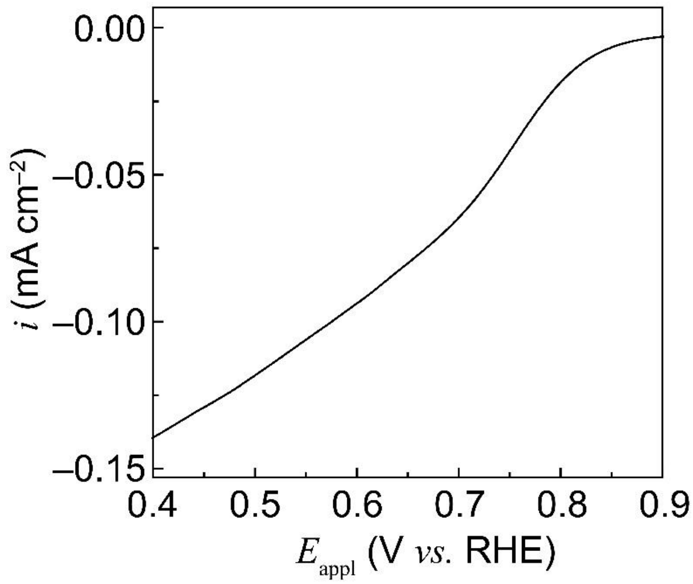

Fig. S4. Linear scan voltammetry (LSV) measurement on Pt-coated wire array electrodes from 0.9 V *vs.* RHE to 0.4 V *vs.* RHE. The calculated Tafel slope was 110 mV/decade and the estimated exchange current density was estimated to be 3×10^{-7} mA/cm^2. RHE, reversible hydrogen electrode.

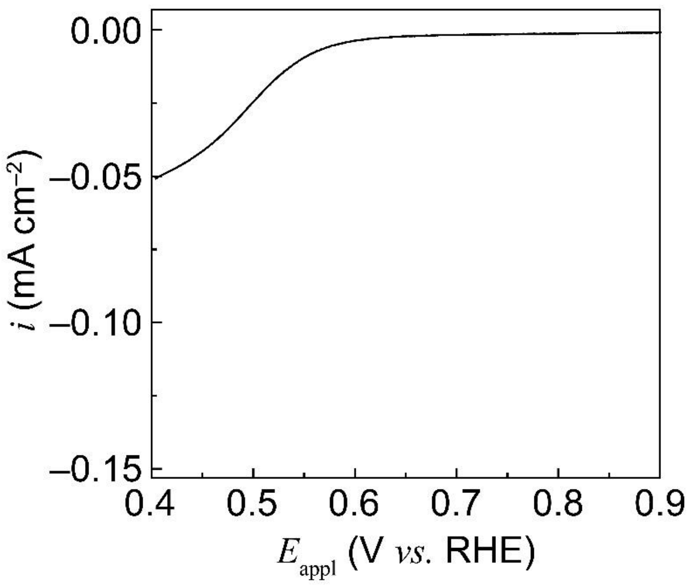

Fig. S5. Linear scan voltammetry (LSV) measurement on Au-coated wire array electrodes from 0.9 V *vs.* RHE to 0.4 V *vs.* RHE. The fitted slope (176 mV/decade) supported electron transfer number and exchange current density of two-electron oxygen reduction reaction (ORR) and four-electron ORR on Au-coated wire array electrodes in simulation.

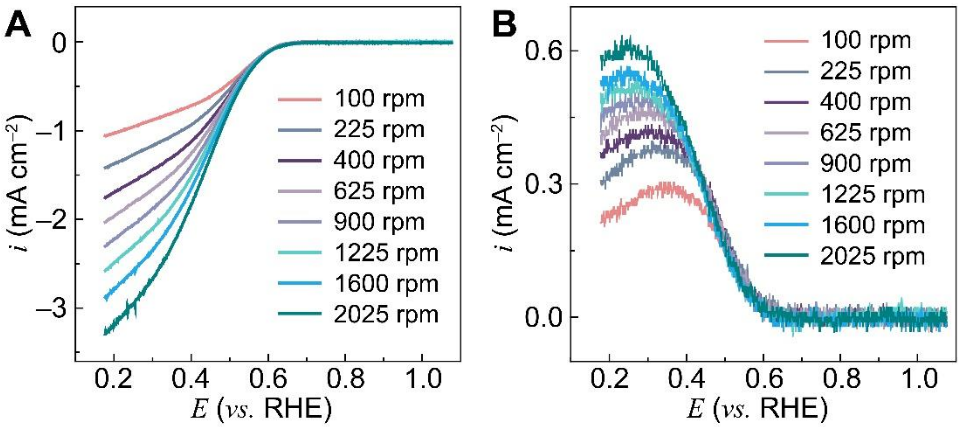

Fig. S6. Rotating ring-disk electrode (RRDE) voltammetry on Au-coated working electrodes. (*A*) Voltammetry measurement on an Au-coated disk electrode. (*B*) Voltammetry measurement on a Pt ring electrode. The electrochemical potential applied on the Pt ring electrode was 1.9 V *vs.* RHE during the measurement.

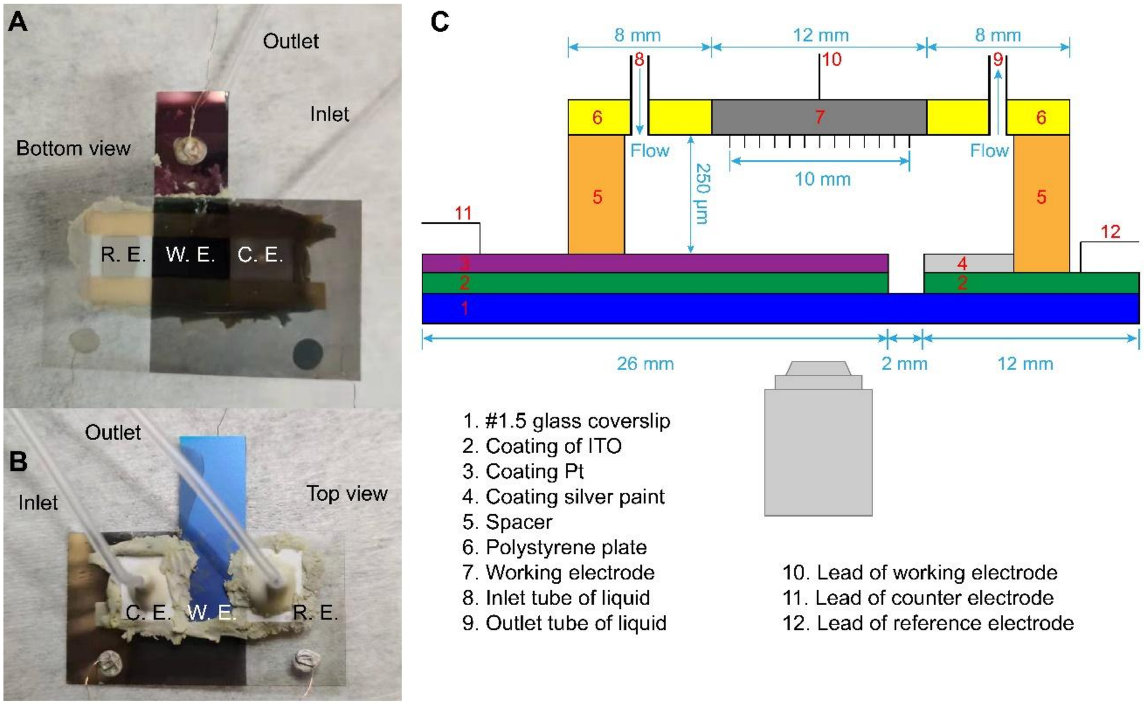

Fig. S7. Photo of a fluidic device for a confocal microscope. (*A*) The bottom view of the fluidic device. (*B*) The top view of the fluidic device. (*C*) The device setup for confocal microscope. The objective lens is shown here in a setting of an inverted confocal laser scanning microscope. W. E., working electrode. C. E., counter electrode. R. E., reference electrode.

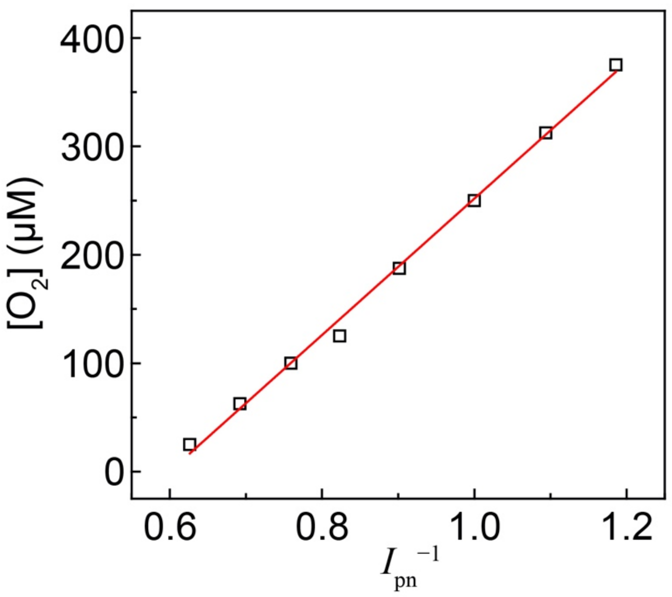

Fig. S8. Plot of inverse of the measured normalized phosphorescence intensity I_{pn}^{-1} under various O_2 concentration. The I_{pn} of tris(1,10-phenanthroline)ruthenium(II), $Ru(Phen)_3^{2+}$ (Phen = 1,10-Phenanthroline) from 600 nm to 640nm (see "Materials and Methods" section) was measured under different O_2 concentration, which functioned as the standard curve for $[O_2]$ calibration.

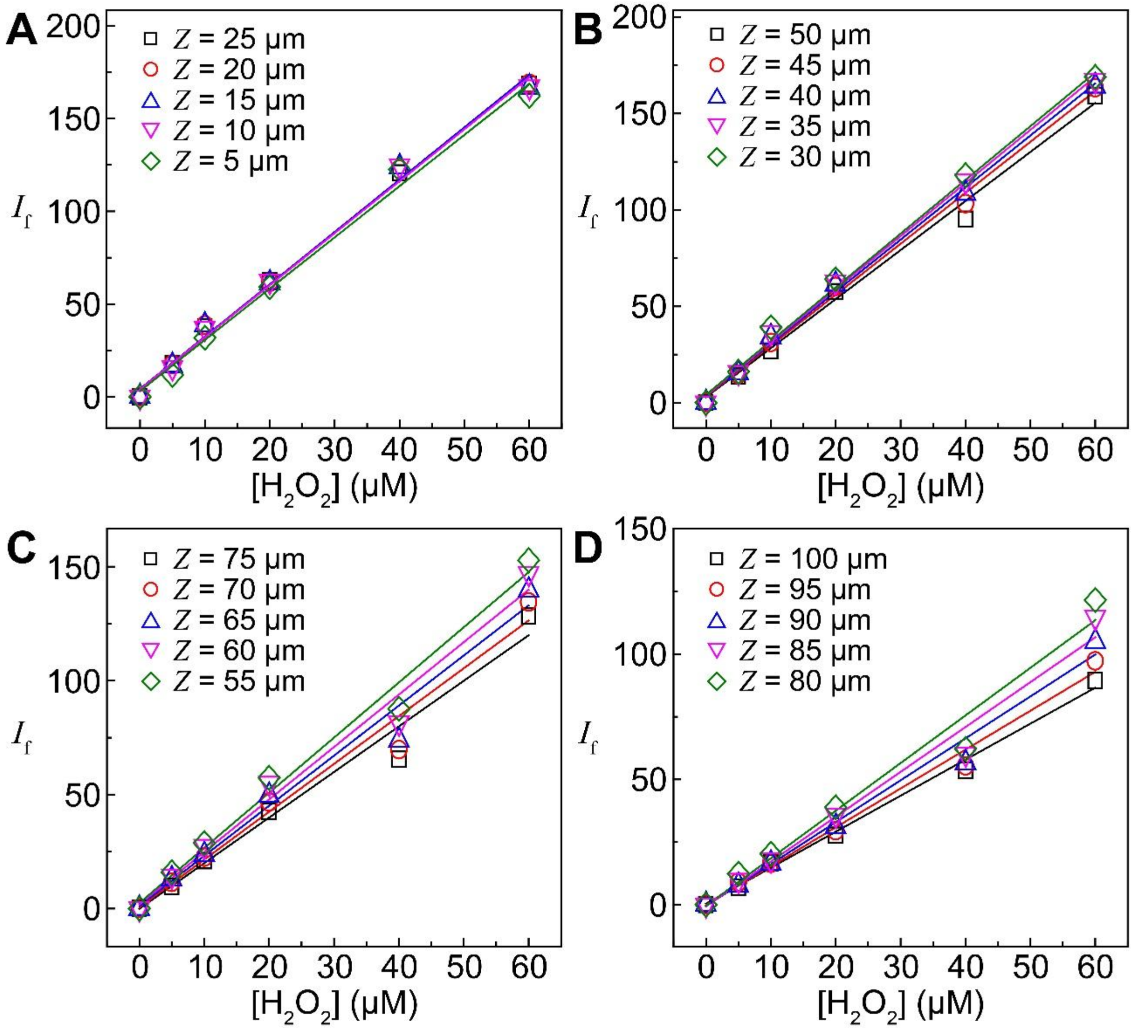

Fig. S9. Plot of measured fluorescence intensity (I_f) under various H_2O_2 concentration on Au-coated wire array electrodes with morphology of ***k*** = (15, 4, 50). The local fluorescence intensity of resorufin from 590 nm to 650 nm I_f (see "Materials and Methods" section) was measured under different H_2O_2 concentration. *Z* is the distance between measured region and the base of the wire array. The relationship between I_f and local H_2O_2 concentration ($[H_2O_2]$) was used for $[H_2O_2]$ calibration on Au-coated wire array electrodes. Morphological vector ***k*** = (*P*, *D*, *L*) presented the wire arrays' periodicity (*P*), diameter (*D*), and length (*L*), respectively, and unit is μm. (*A*) Calibration curves for region from *Z* = 5 μm to 25 μm. (*B*) Calibration curves for region from *Z* = 30 μm to 50 μm. (*C*) Calibration curves for region from *Z* = 55 μm to 75 μm. (*D*) Calibration curves for region from *Z* = 80 μm to 100 μm.

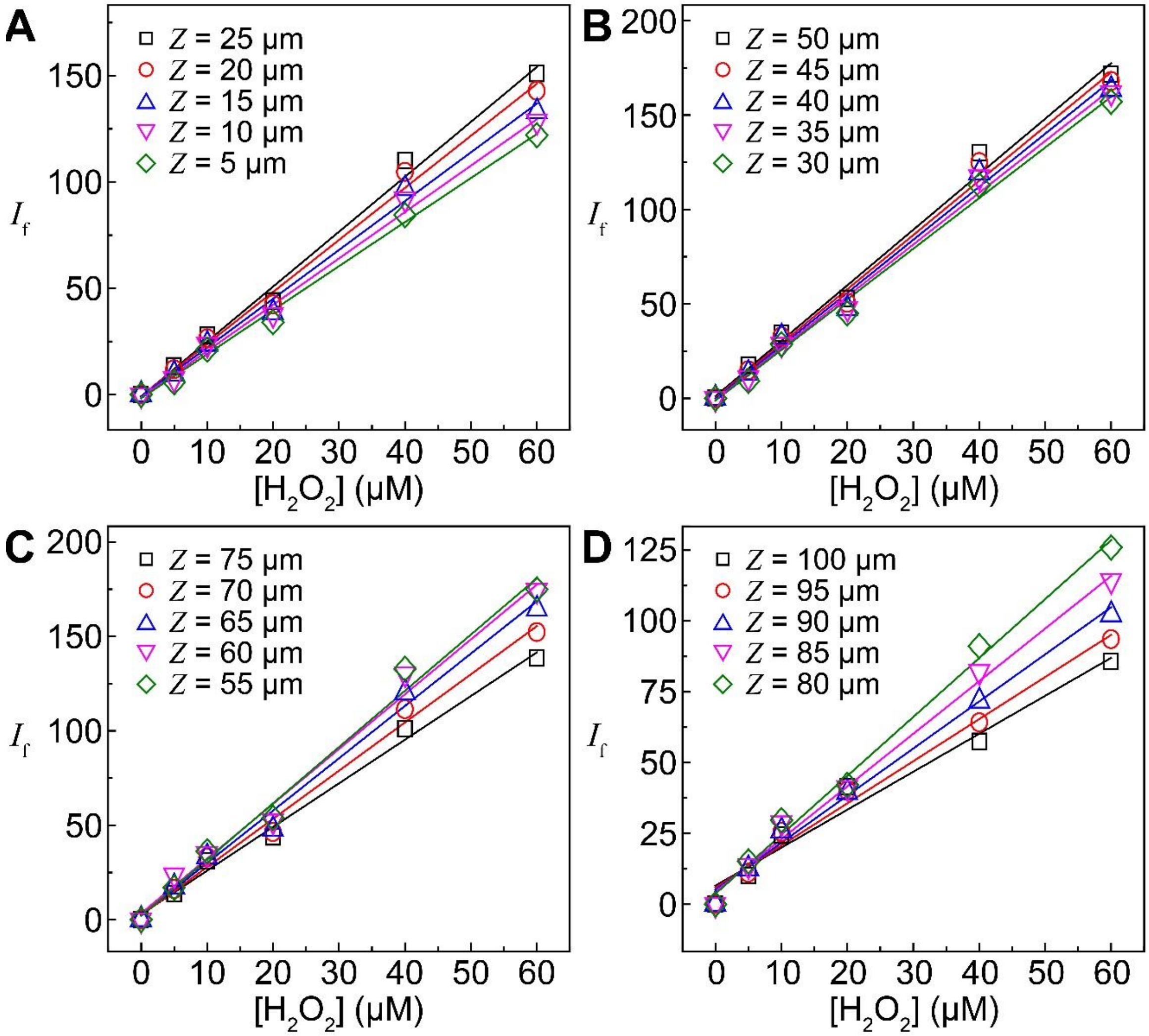

Fig. S10. Plot of measured I_f under various H_2O_2 concentration on Au-coated wire array electrodes with morphology of ***k*** = (30, 3, 50). The local fluorescence intensity of resorufin from 590 nm to 650 nm I_f (see "Materials and Methods" section) was measured under different H_2O_2 concentration. *Z* is the distance between measured region and the base of the wire array. The relationship between I_f and [H_2O_2] was used for [H_2O_2] calibration on Au-coated wire array electrodes. (*A*) Calibration curves for region from *Z* = 5 μm to 25 μm. (*B*) Calibration curves for region from *Z* = 30 μm to 50 μm. (*C*) Calibration curves for region from *Z* = 55 μm to 75 μm. (*D*) Calibration curves for region from *Z* = 80 μm to 100 μm.

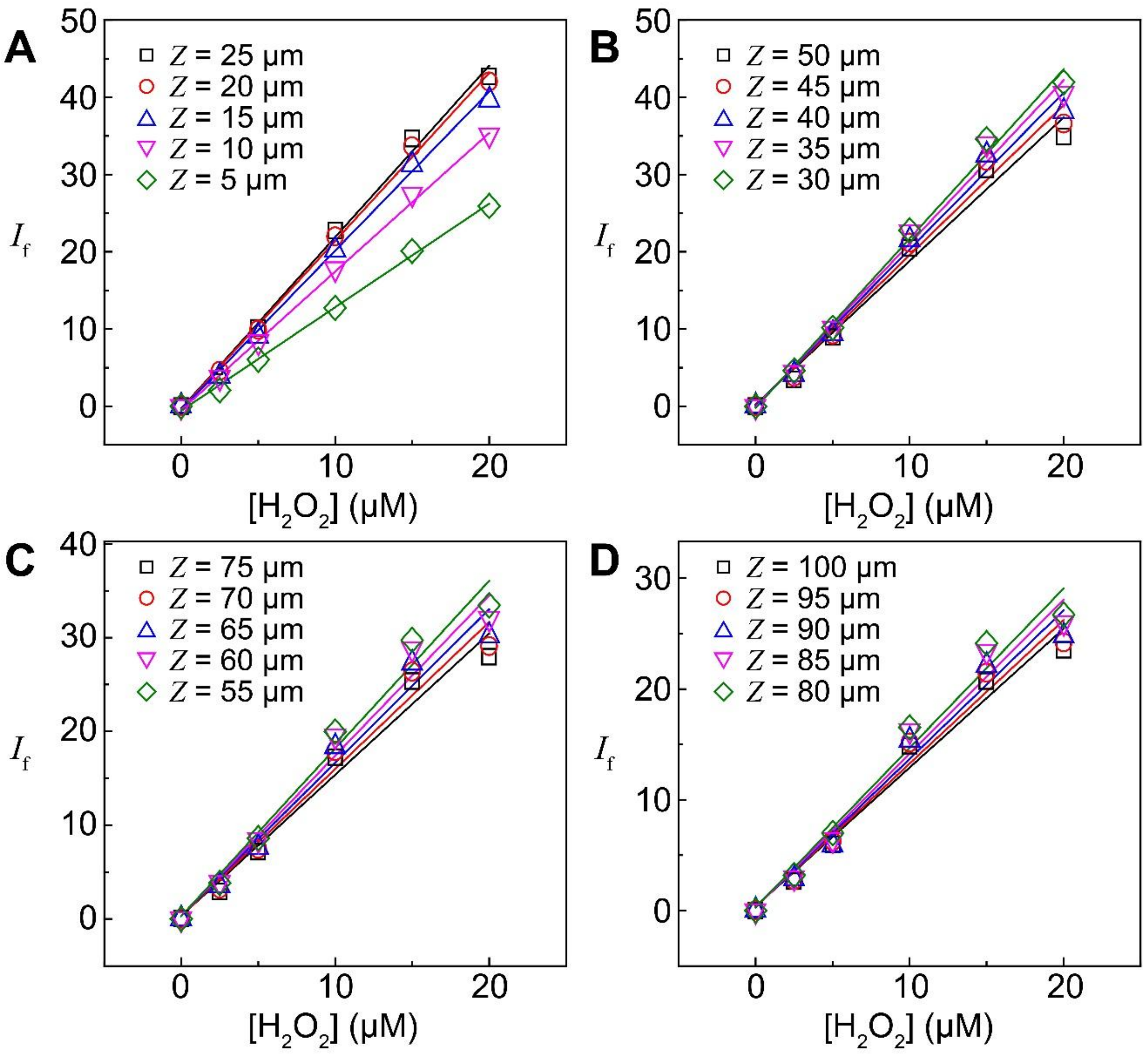

Fig. S11. Plot of measured I_f under various H_2O_2 concentration on Au-coated wire array electrodes with morphology of ***k*** = (15, 4, 20). The local fluorescence intensity of resorufin from 590 nm to 650 nm I_f (see "Materials and Methods" section) was measured under different H_2O_2 concentration. *Z* is the distance between measured region and the base of the wire array. The relationship between I_f and [H_2O_2] was used for [H_2O_2] calibration on Au-coated wire array electrodes. (*A*) Calibration curves for region from *Z* = 5 μm to 25 μm. (*B*) Calibration curves for region from *Z* = 30 μm to 50 μm. (*C*) Calibration curves for region from *Z* = 55 μm to 75 μm. (*D*) Calibration curves for region from *Z* = 80 μm to 100 μm.

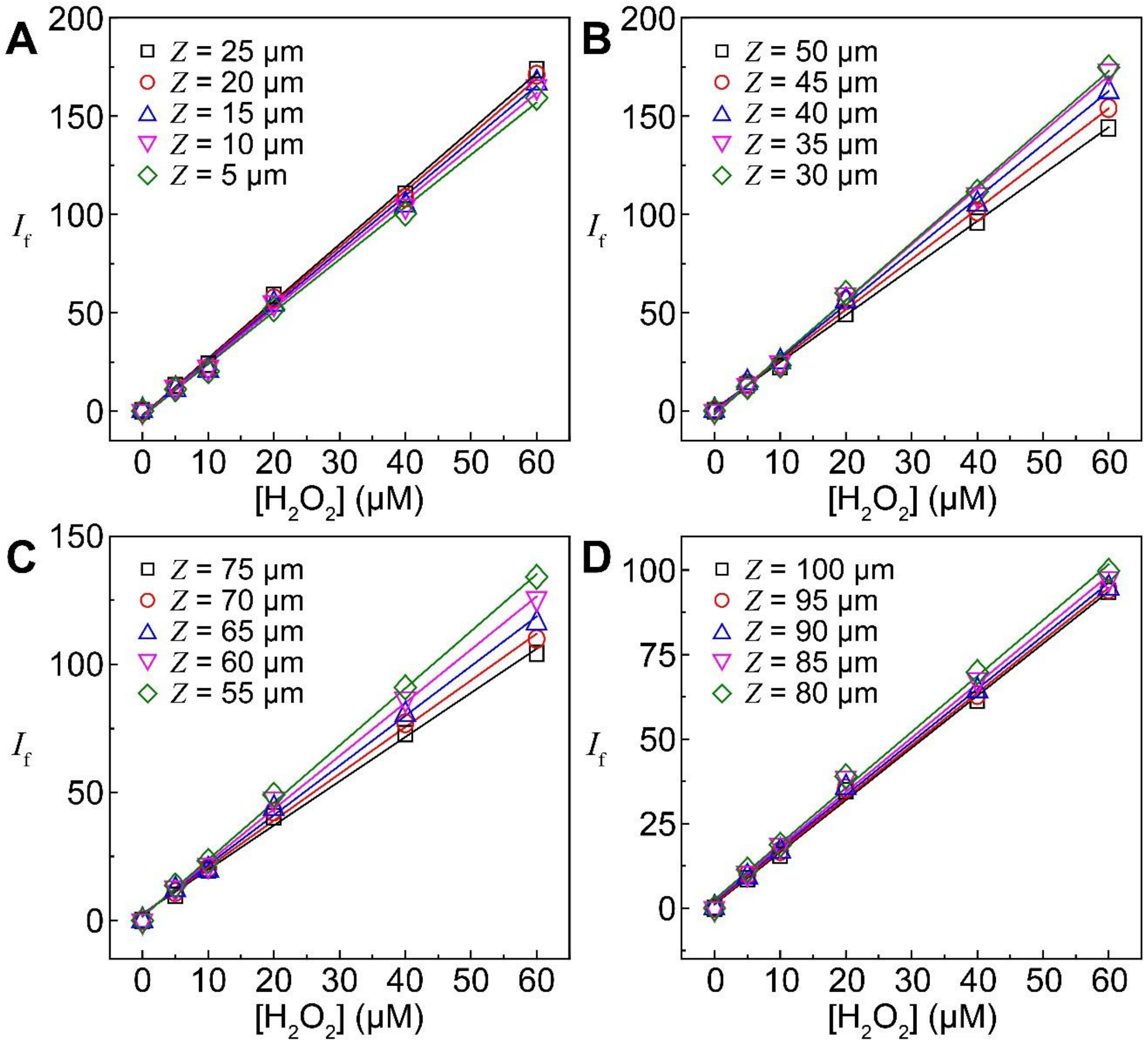

Fig. S12. Plot of measured I_f under various H_2O_2 concentration on Au-coated wire array electrodes with morphology of ***k*** = (17, 3, 30). The local fluorescence intensity of resorufin from 590 nm to 650 nm I_f (see "Materials and Methods" section) was measured under different H_2O_2 concentration. *Z* is the distance between measured region and the base of the wire array. The relationship between I_f and [H_2O_2] was used for [H_2O_2] calibration on Au-coated wire array electrodes. (*A*) Calibration curves for region from *Z* = 5 μm to 25 μm. (*B*) Calibration curves for region from *Z* = 30 μm to 50 μm. (*C*) Calibration curves for region from *Z* = 55 μm to 75 μm. (*D*) Calibration curves for region from *Z* = 80 μm to 100 μm.

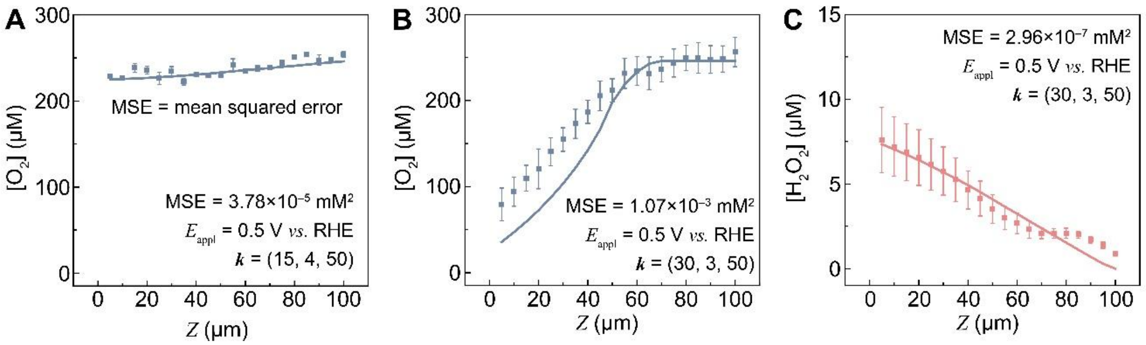

Fig. S13. Simulated and experimental O_2 gradient and H_2O_2 gradient on Pt/Au-loaded wire array electrodes under E_{appl} = 0.5 V *vs.* RHE. (*A*) Simulated and experimental O_2 gradient on Au-loaded wire array electrodes with $\boldsymbol{k}$ = (15, 4, 50). (*B*) Simulated and experimental O_2 gradient on Pt-loaded wire array electrodes with $\boldsymbol{k}$ = (30, 3, 50). (*C*) Simulated and experimental H_2O_2 gradient on Au-loaded wire array electrodes with $\boldsymbol{k}$ = (30, 3, 50). All the dots are experimental data and all the lines are corresponding simulated gradients. Although O_2 gradients on Au-loaded wire array electrodes (a) are small perturbations (around 0.2 μM/μm), the simulation still achieved low mean squared error. MSE, mean squared error. Error bars represent standard deviations across multiple separate measurements in the device ($n \geq 3$).

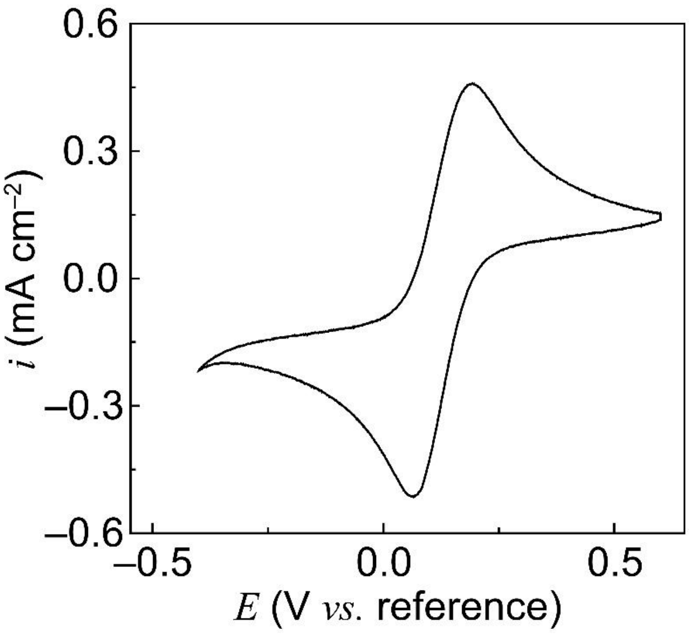

Fig. S14. Cyclic voltammetry measurement in ZoBell's solution using Ag-pseudo reference electrode. Besides, a platinum wire and a glassy carbon electrode were used as the counter electrode and the working electrode respectively. The cyclic voltammetry measurement was conducted from 0.6 V *vs.* reference to –0.4 V *vs.* reference at a scan rate of 20 mV/s.

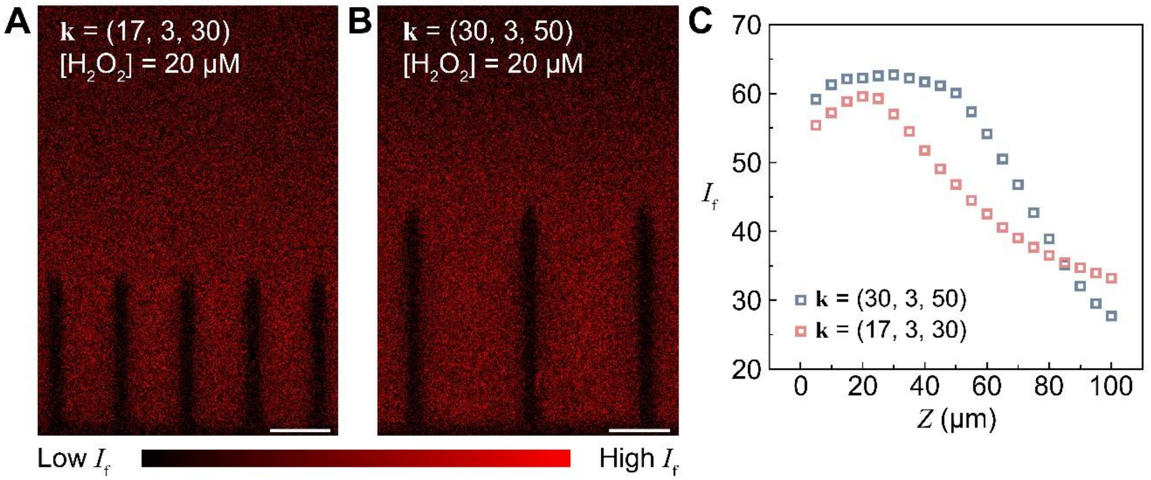

Fig. S15. Fluorescence intensity I_f measurement under different morphologies. (*A*) Confocal image with ***k*** = (17, 3, 30) when [H_2O_2] = 20 μM (*B*) Confocal image with ***k*** = (30, 3, 50) when [H_2O_2] = 20 μM (*C*) I_f mapping comparison between 2 morphologies when [H_2O_2] = 20 μM, suggesting I_f is related to both morphology and location. Scale bar, 15 μm.

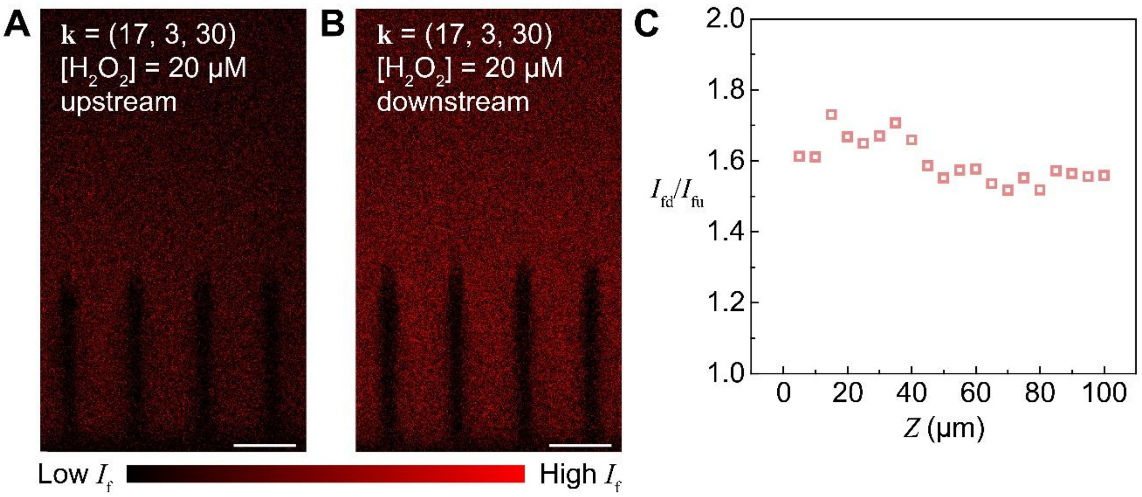

Fig. S16. I_f difference between the upstream and downstream of the wire array electrode. (*A*) Confocal image with ***k*** = (17, 3, 30) when [H_2O_2] = 20 μM taken at the upstream of the wire array electrode (*B*) Confocal image with ***k*** = (17, 3, 30) when [H_2O_2] = 20 μM taken at the downstream of the wire array electrode (*C*) The ratio between fluorescence intensity at the downstream and fluorescence intensity at the upstream (I_{fd}/I_{fu}) at different location. The average ratio is around 1.6. The travel time from upstream to downstream was calculated as 0.84 s, suggesting I_f will increase by 1.9 times each second.

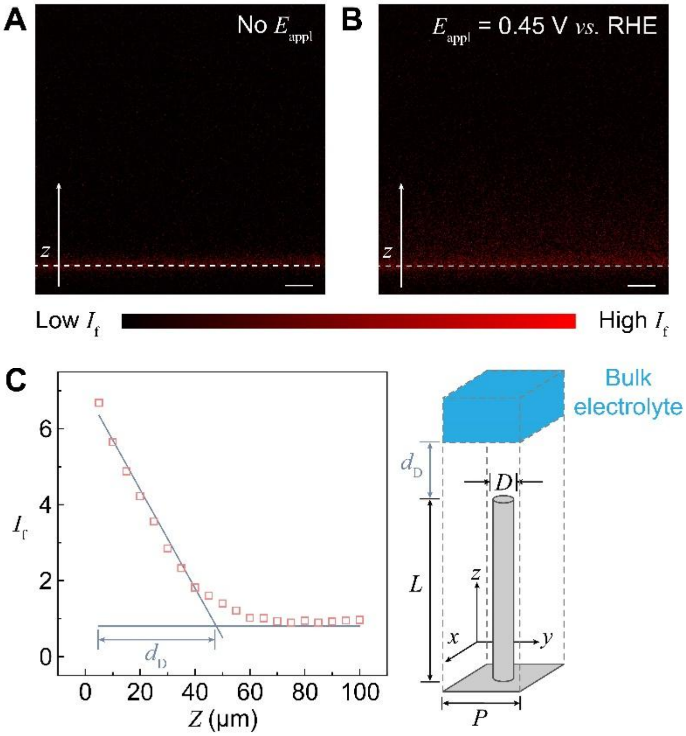

Fig. S17. Fluorescence mapping on Au-coated planar electrode. (*A*) The fluorescence intensity ($\boldsymbol{I_f}$) mapping on Au-coated planar electrode without E_{appl} (*B*) The I_f mapping on Au-coated planar electrode when E_{appl} = 0.45 V *vs.* RHE (*C*) Plotting of local I_f against the local distance from the base of the electrode (*Z*) on Au-coated planar electrode with E_{appl} = 0.45 V *vs.* RHE. The diffusion layer thickness, d_D, is determined by the intersection of the two fitted red lines. On Au-coated electrodes, d_D was 50 μm. Scale bar, 15 μm.

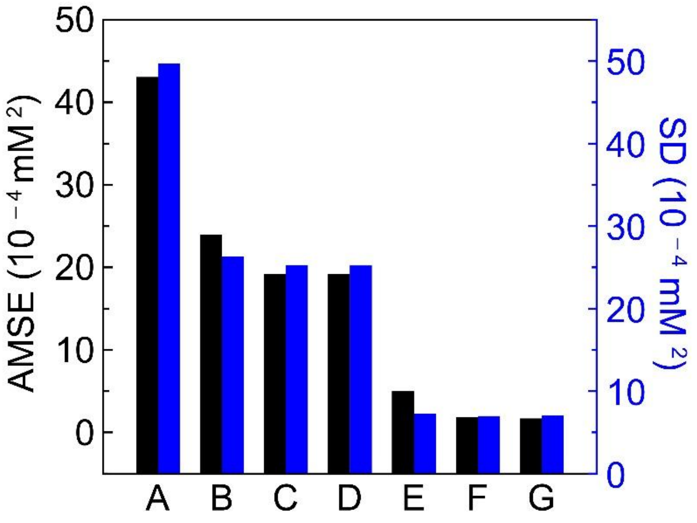

Fig. S18. Machine learning model selection for O_2 gradient prediction on Pt-loaded wire array electrodes. The average mean square error (AMSE) and standard deviation (SD) of mean square error (MSE) in various machine learning approaches for O_2 gradient prediction on Pt-loaded wire array electrodes (A is Random Forest Regressor, B is Support Vector Regression, C is Bayesian Ridge Regression, D is Stochastic Gradient Descent, E is Adaboost Regressor, F is K-Nearest Neighbors Regressor, G is Multiple-layer Perceptron Neural Networks).

Table S1: Phosphate-buffered saline (PBS)

Component	Concentration (g/L)
NaCl	8
KCl	0.2
Na_2HPO_4	1.44
KH_2PO_4	0.24
